# Open‐source bioreactor delivers electrical and perfusion stimulation supporting 3D cardiac engineered tissue maturation

**DOI:** 10.1002/btm2.70145

**Published:** 2026-04-13

**Authors:** Gregory Reid, Stefano Gabetti, Antonio Sileo, Deborah Fusco, Giuseppe Isu, Diana Massai, Giulia Milan, Anna Marsano

**Affiliations:** ^1^ Department of Biomedicine University of Basel and University Hospital of Basel Basel Switzerland; ^2^ Department of Plastic and Hand Surgery University Hospital of Zürich Zürich Switzerland; ^3^ Department of Mechanical and Aerospace Engineering and PolitoBIOMed Lab Politecnico di Torino Turin Italy

**Keywords:** bioreactor, cardiomyocyte, electrical stimulation, in vitro cardiac models, perfusion, tissue engineering

## Abstract

Cardiac tissue engineering requires control over physical stimuli, such as mechanical and electrical, to promote the maturation and functionality of cardiomyocytes. While perfusion bioreactors and electrical stimulation systems have been employed before, their synergistic impact, specifically when using direct perfusion in soft hydrogel environments, remains underexplored. We developed a cost‐effective, modified perfusion bioreactor that is commercially available, to integrate electrical stimulation to support the culture of fibrin‐based cardiac constructs. The system delivers continuous unidirectional flow (0.3 mL/min) and controlled electrical pulses (3 V/cm, 1 Hz), with validated flow and field uniformity via computational fluid dynamics and finite element analysis simulations. Neonatal rat cardiac cell‐based constructs were cultured either under static or perfusion conditions in presence or not of electrical stimulation. Cellular outcomes were evaluated by immunofluorescence, gene expression, and live‐cell functional analyses. Perfusion significantly improved cell retention and cardiomyocyte yield, while electrical stimulation promoted cardiomyocyte elongation, sarcomere organization, and maturation. The combination of perfusion and electrical stimulation led to the highest proportion of mature cardiomyocytes (51.4%), significantly outperforming perfusion alone (12.3%), static combined with electrical stimulation (22.2%), and static alone (7.5%). A reduced fibroblast activation was observed, along with enhanced tissue remodeling, as shown by increased extracellular matrix density and upregulation of remodeling genes. Functionally, constructs under perfusion with electrical stimulation exhibited the lowest excitation thresholds, highest maximum capture rates, and the greatest contraction displacement (2.2 ± 0.49 V, 4.2 ± 0.71 Hz, 13.35 ± 3.6 μm respectively), confirming superior functional performance. Our integrated bioreactor system enables efficient culture of soft 3D cardiac tissues and demonstrates that combining perfusion with electrical stimulation synergistically enhances cardiomyocyte maturation, construct remodeling, and functional performance. Beyond being a promising tool, this platform represents a powerful and scalable solution for cardiac tissue engineering, with potential for applications in disease modeling, drug screening, and regenerative medicine.

**Translational Impact Statement**

In this work, we present a novel, open‐source adaptation of a commercially available bioreactor that enables direct perfusion through soft hydrogels, combined with electrical stimulation, to support the maturation of engineered cardiac tissues. Our data show a significant improvement of the *structural organization and functional maturation* of engineered cardiac tissues. While this proof‐of‐concept study demonstrates clear benefits for cardiac tissue engineering, designed to be *cost‐effective, customizable, and open‐source*, making it broadly applicable also for other soft tissue models requiring the integration of multiple biophysical stimuli.

## 1 INTRODUCTION

The worldwide burden of cardiovascular disease (CVD) has increased dramatically, with accredited deaths risen by 60% over the past 30 years. Low‐ and middle‐income countries are particularly affected, with four in five deaths caused by CVD.^1^ For patients with end‐stage heart disease, no cure exists yet, and current treatments only provide symptomatic relief and slow disease progression.^2^ Tissue engineering offers a promising approach for investigating cellular physiology, modeling disease, and developing new treatments for heart failure. However, a great challenge in cardiomyocyte‐based models and therapies is achieving sufficient cellular maturation. Induced or embryonic pluripotent stem cells or neonatal rodent cells share an immature phenotype,^3^ which must be overcome for therapeutic applications.^4^ Immature cells pose significant risks, including arrythmias due to improper electrical coupling,^5^ premature cell death or dedifferentiation coupled possibly even with tumorigenicity.^6^ To promote cardiac maturation, various strategies have been explored, including prolonged culture time, co‐culturing with other cell types, hormonal additives, and electrical stimulation.^5^, ^7^ Early electrical stimulation^8^ has been shown to increase the electrical handling capabilities and the creation of a functional syncytium. Further, cell alignment and coupling, ultrastructural organization and mitochondrial development are improved.^9^, ^10^ Key parameters such as voltage, frequency and pulse duration can be adjusted to optimize outcomes.^11^ However, direct electrode contact with the culture medium may release toxic byproducts, which necessitates a perfusion system to dilute harmful substances. Perfusion is also essential for generating thicker engineered tissues.^12^ Cardiomyocytes have high metabolic demands and are particularly sensitive to direct shear stress, posing a limitation for *in vitro* cardiac tissue engineering. Nutrient and oxygen diffusion is typically limited to distances below 300 μm, making it challenging to sustain thicker engineered tissues.^13^ In cardiac tissue engineering approaches, the integration of multiple stimuli, particularly electrical stimulation, might further contribute to the accumulation of waste products, including those generated by electrode oxidation.^10^ To mitigate the accumulation of metabolic and possibly non‐metabolic waste products and to maintain a physiologically relevant extracellular environment, multiple strategies have been explored. These include frequent cell media changes, possibly increasing risks of contaminations, and bioreactor‐based perfusion, which have been tested to remove the extra‐cellular surrounding of waste byproducts and maintain an equally distributed physiological cell‐surrounding.^14^, ^15^, ^16^, ^17^ While bioreactors can integrate multiple stimuli, such as electrical and mechanical stimulations,^18^, ^19^, ^20^ even under real‐time assessment,^17^, ^21^, ^22^ most rely on indirect perfusion or permeable porous scaffolds.^23^, ^24^, ^25^ To date, there exists no reported system employing cardiomyocyte‐specific hydrogels^26^ cultured under direct perfusion in combination with electrical stimulation. To minimize direct shear stress exposure to cardiomyocytes,^27^, ^28^ engineered tissues often incorporate porous scaffolds or hydrogels with internal channels.^29^ Hydrogels^30^ are particularly advantageous due to their tunable mechanical and biochemical properties, compatible with direct casting or 3D printing,^31^ biocompatibility, and capability to support close cell–cell interactions, cellular remodeling, and controlled degradation. Fibrin‐based hydrogels, often used in cardiac tissue engineering,^19^, ^32^, ^33^ provide a tested clinical safety profile and allow for controlled degradation. Unlike previous studies combining electrical stimulation with medium perfusion through porous scaffolds or indirect perfusion channels embedded with a fibrin hydrogel matrix,^34^, ^35^ here we propose a platform that delivers direct perfusion through an array of channels embedded within a fibrin hydrogel matrix.^26^ While porous scaffolds, restrict cells to surface adhesion and expose them to heterogeneous interstitial flow, fibrin hydrogels enable full cellular encapsulation, supporting robust three‐dimensional cell–cell interactions and tissue remodeling. By introducing perfusable channels, our approach overcomes the diffusion limitations inherent to hydrogels while avoiding the excessive shear stress associated with forced interstitial perfusion, thereby providing a more protective and physiologically relevant microenvironment for cardiomyocyte culture under electrical stimulation.

**FIGURE 1:**
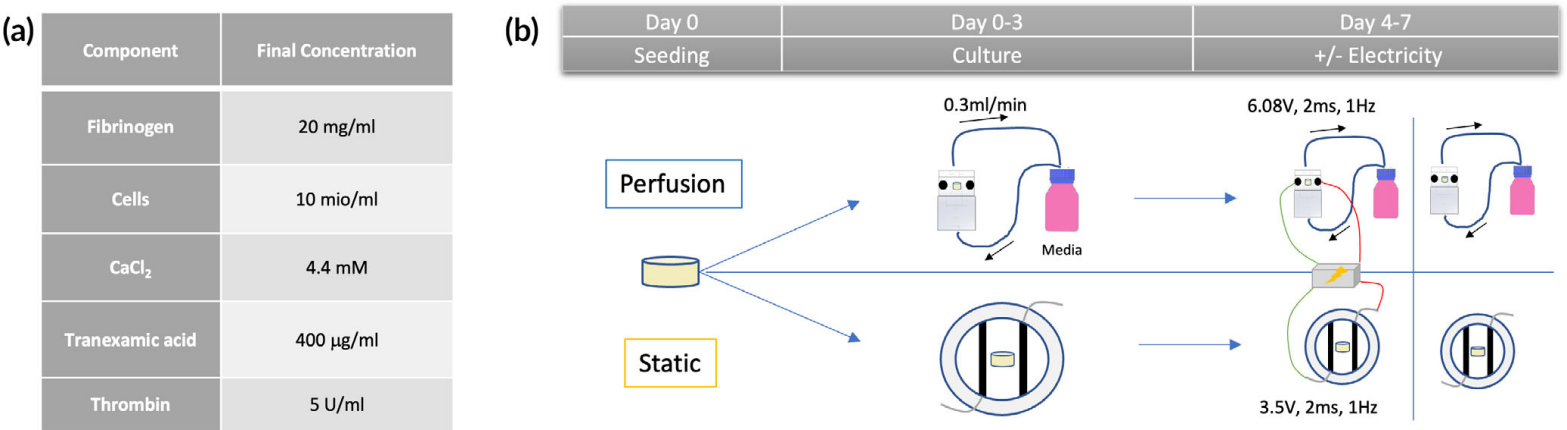
Study experimental plan. (a) Hydrogel composition list. The components are given as the concentrations found in the final solution of 100 μL. (b) Experimental time plan. At day 0, cell‐laden constructs were placed either in a perfusion culture chamber (top row) or in a static culture system (bottom row). In the perfusion group, unidirectional culture media flow was initiated immediately after seeding at 0.3 mL/min. On day 4, electric stimulation was applied to the intervention group: 6.08 V, 2 ms and 1 Hz for the perfusion group (top row) and 3.5 V, 2 ms and 1 Hz for the static group (bottom row).

In this study, we introduce a new open‐source bioreactor design that allows for direct perfusion of fibrin‐based hydrogels under electrical stimulation. As a proof‐of‐concept, neonatal rat cardiomyocytes were seeded as an 8 mm × 2 mm hydrogel and perfused for 7 days, with electrical stimulation applied after day 3 (Figure 1). We hypothesized that combining direct perfusion with electrical stimulation for culture of fibrin gel‐based cardiac constructs will enhance cell–cell interactions and tissue remodeling, and improve cardiac functionality and maturation.

## 2 RESULTS

### 2.1 Development of a bioreactor with a continuous direct perfusion flow combined with electrical stimulation

**FIGURE 2:**
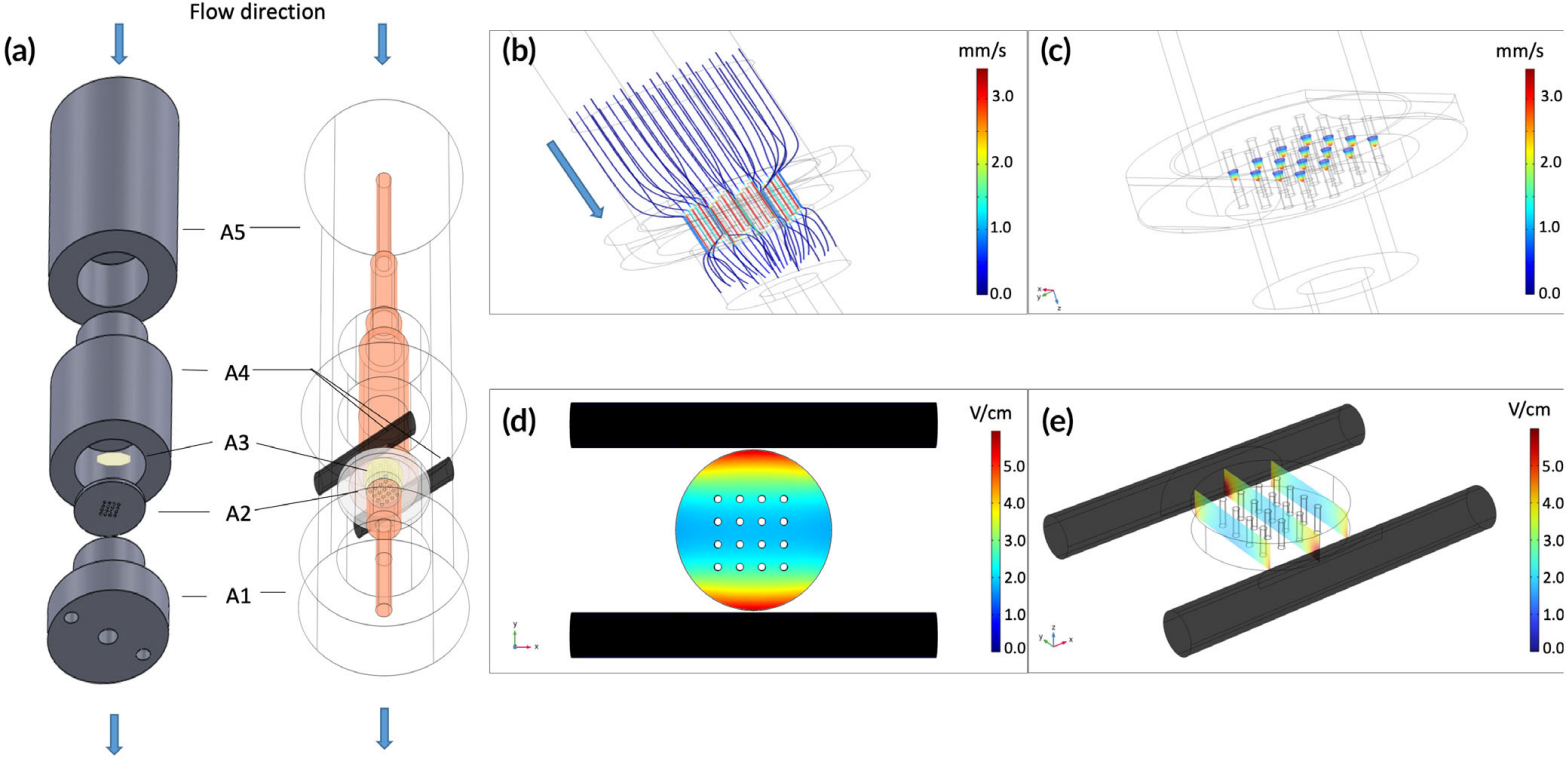
Bioreactor design and characterization. (a) Schematic representation of the bioreactor chamber showing the lid (A1), PDMS membrane with holes (A2), scaffold (A3, yellow), custom PDMS insert with carbon rods (A4) and bottom (A5). Culture medium is perfused unidirectionally through the chamber (blue arrows) and passes through the fluid channels within the construct (orange). (b) Simulated flow streamlines within the culture chamber. (c) Close‐up representation of the perfusion channel showing the velocity distribution (mm/s); the direction of the flow is indicated by blue arrow. (d, e) Simulated electric field magnitude through the construct throughout the horizontal (d) and the vertical (e) plane.

The main design consideration was to modify an existing commercially available perfusion bioreactor system, in order to add electrical stimulation using a cost‐efficient and previously established bioreactor set‐up (Figures 2a and S1). Further requirements comprised the ability to easily modify the newly designed PDMS inset, as well as include the capability to perfuse soft, fibrin‐based hydrogels. A hole punch would deliver 16 equally spaced holes of 0.5 mm diameter each to allow for equal distribution of flow.^26^ Because the cells are suspended in the hydrogel, classic shear stress calculations^12^ pertaining to cardiomyocytes under perfusion do not apply.

Computational fluid dynamics simulations allowed for characterization of culture medium flow within the culture chamber at the imposed flow rate of 0.3 mL/min.^36^, ^37^ In detail, the flow streamlines within the culture chamber reveal the absence of recirculation regions before and after the hydrogel construct (Figure 2b). The culture medium flow was equally distributed among the construct channels and fully developed, as reflected by the parabolic velocity profile, reaching a maximum velocity of 3.2 mm/s in each channel (Figures 2c and S1). The wall shear stress inside the construct channels resulted uniformly distributed, with an average value of 17.4 mPa (0.174 dyne/cm^2^) (Figure S1). The corresponding pressure drop along the channel was 0.44 Pa (4.4 dyne/cm^2^).

A custom‐made stimulator^38^ delivered rectangular pulses of 1 Hz, 2 ms duration and 6.08 V, resulting in an electric field of 3 V/cm and parallel electric field throughout the center of the hydrogel construct.

The distributions of the electric field and of the current density within the hydrogel construct were characterized by performing an electric field finite element analysis. When applying a voltage of 6.08 V, close to the electrodes, the electric field magnitude is about 6 V/cm, while the central region of the construct is exposed to an almost uniform electric field value of 3 V/cm (Figure 2d,e). The direction of the current is well aligned along the electrode–electrode direction, and due to the uniformity of the electric field, the current density is uniform in the central region of the construct with an absolute value of 34 mA/cm^2^ (Figure S1). A photographic representation can be found in Figure S2.

### 2.2 Cellular characterization

**FIGURE 3:**
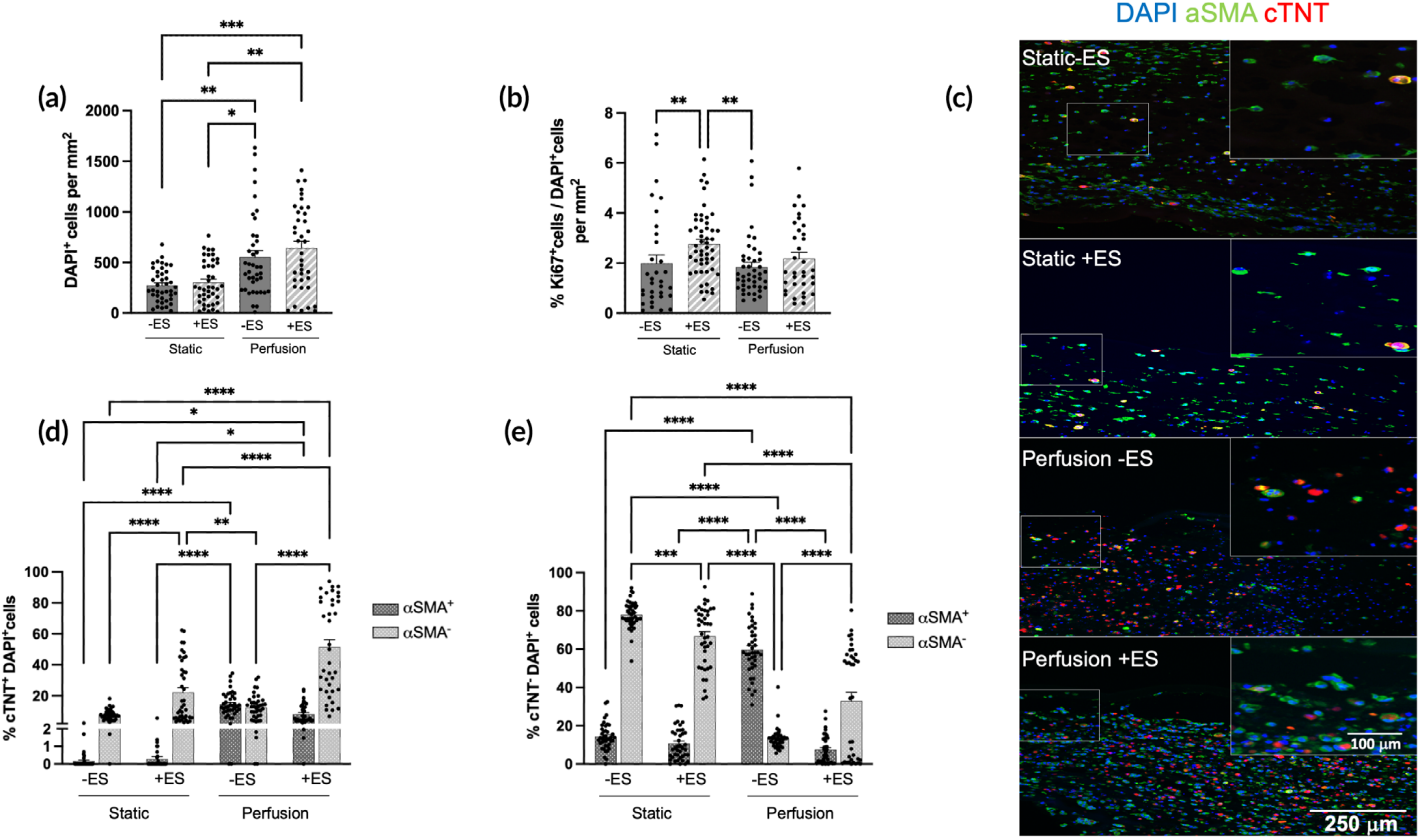
Cellular characterization analyses. (a) Viable cell quantification based on DAPI staining per mm^2^. (b) Quantification of Ki67^+^ expressing cells, relative to the number of cells expressing DAPI (%) per mm^2^. (c) Immunofluorescence staining of cardiac constructs. DAPI stained in blue, αSMA stained in green, cTNT stained in red. (d) Quantification of mature cardiomyocytes (cTNT^+^αSMA^−^) and immature cardiomyocytes (cTNT^+^ αSMA^+^). (e) Quantification of activated fibroblasts (cTNT^−^ αSMA^+^) and fibroblasts (cTNT^−^ αSMA^−^). Both (d) and (e) are relative to the number of cells expressing DAPI (%) per mm^2^. All data are represented as mean ± SEM. Comparisons were performed using a two‐way ANOVA and a Tukey's post‐hoc test. (*p < 0.05, **p < 0.01, ***p < 0.001, ****p < 0.0001). All histological and immunofluorescent analyses were performed on a minimum of two independent experiments.

Immunofluorescent analysis revealed a significantly higher total number of cells in both perfusion groups after 7 days of culture compared to the static conditions, despite all groups starting with an equal number of seeded cells (Figure 3a). Quantification of the Ki67^+^ cells (Figure 3b) indicated that the overall cell proliferation remained low across all conditions, with less than 3% of cells expressing Ki67 after 7 days. However, a modest increase in proliferating cells was observed in both +ES groups, with a significant increase in the Static +ES group compared to both non‐stimulated groups (exemplary staining in Figure S3).

Perfusion culture also resulted in a higher proportion of cardiomyocytes than static conditions, as demonstrated by cardiac troponin T (cTNT) staining (Figure S3). To further investigate whether perfusion culture, with or without electrical stimulation, promotes cardiac maturation or induces fibroblast‐to‐myofibroblast transition, immunofluorescent analysis was performed for cardiomyocytes (cTNT) and myofibroblast (αSMA) markers, respectively (Figure 3c). Although cardiomyocytes appeared isolated in all groups, closer spatial proximity between cardiomyocytes was more pronounced in the perfusion groups, especially with ES.

Overall, quantification of all cardiomyocytes (DAPI^+^ and cTNT^+^ cells) revealed a higher presence in both perfusion groups compared to static groups (Figure S3). While cells co‐expressing cTNT and αSMA, indicative of a less mature phenotype, were more frequently observed in the perfusion groups compared to static conditions, the relative percentage of cardiomyocytes exhibiting a more mature phenotype (cTNT^+^ and αSMA^−^) was highest in the perfusion group with electrical stimulation (Figure 3d).^39^

Fibroblasts were identified as DAPI^+^ and cTNT^−^ cells. Further classification of the cTnT^−^ and αSMA^+^ subpopulation identified them as activated fibroblasts, also known as myofibroblasts. The highest relative proportion hereof was observed in perfusion without electrical stimulation, which was significantly higher compared to the static groups and the perfusion with ES (Figure 3e). Notably, the perfusion + ES group exhibited the lowest percentages of fibroblasts, both αSMA^+^ and αSMA^−^.

These findings indicate that perfusion results in the highest overall cell retention within the engineered cardiac constructs, especially promoting the cardiomyocyte subpopulation, as well as the fibroblast activation. Furthermore, the combination of electrical stimulation and perfusion appears to support cardiac maturation while reducing fibroblast activation.

### 2.3 Cell elongation and contractile unit analysis

**FIGURE 4:**
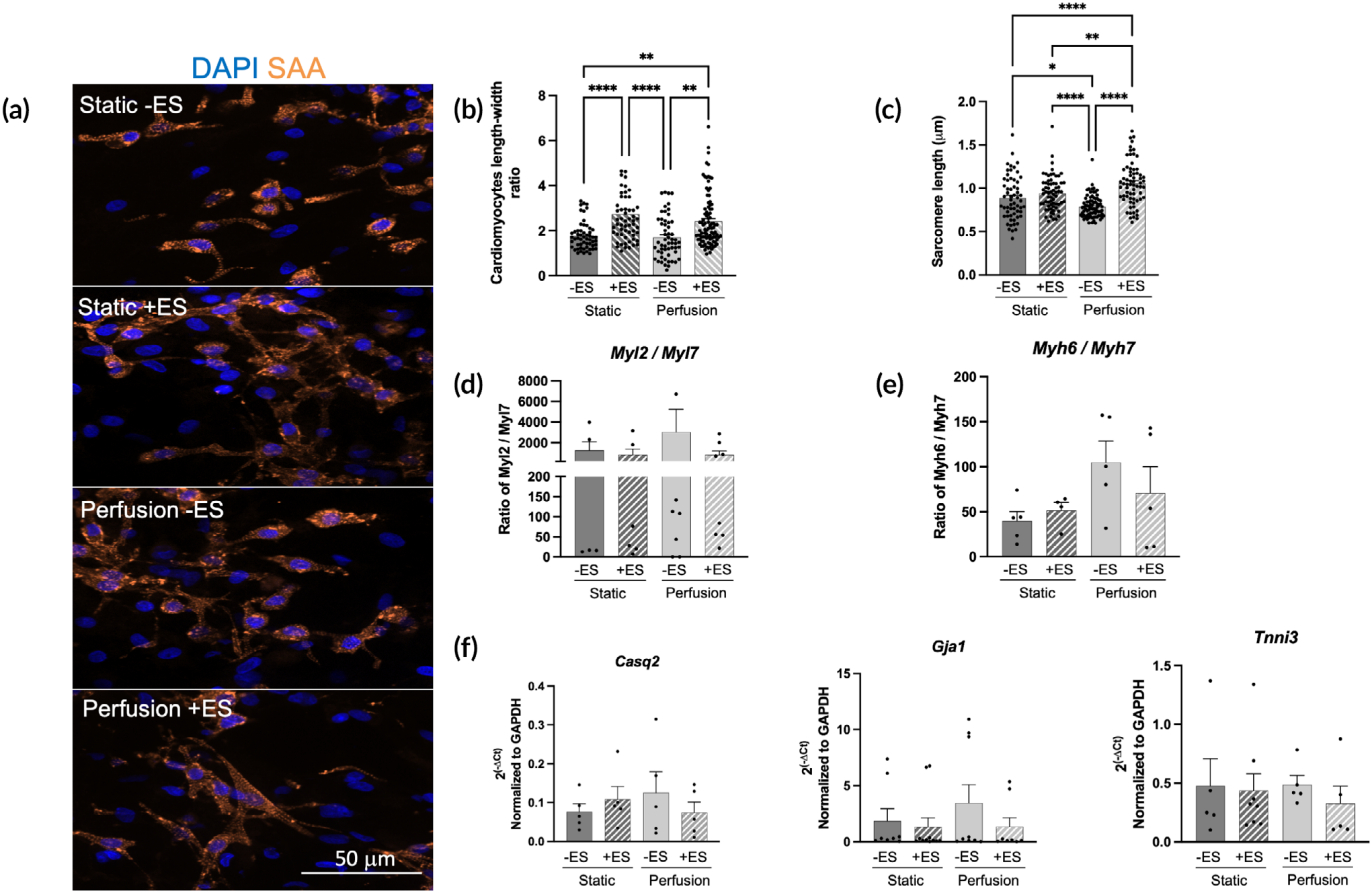
Cardiac maturation: Cell elongation and contractile and electrical unit analyses. (a) Representative images of immunofluorescence for sarcomeric‐α‐actinin stained in orange (SAA). Nuclei are stained with DAPI in blue. (b) Analyses of cardiomyocyte length‐width ratio and (c) average sarcomere length. (d, e) RT‐PCR analysis showing relative expression ratios of myosin chain genes: *Myl2/Myl7* (d) and (e) *Myh6/Myh7*. (f) Gene expression analysis by RT‐PCR of markers involved in electrical conduction (*Casq2, Gja1*) and contractility (*Tnni3*). Comparisons were performed using a two‐way ANOVA test and normalized to *Gapdh* as a housekeeping gene. All data are represented as mean ± SEM. In (a), (d), (e) all ratios are expressed as arbitrary unit. (*p < 0.05, **p < 0.01, ***p < 0.001, ****p < 0.0001). RT‐PCR analysis was performed on a minimum of four samples from a minimum of two independent experiments.

Immunofluorescence analysis for sarcomeric α‐actinin (SAA) confirmed that cardiomyocytes under static and perfusion conditions, without ES, appeared to remain more isolated. In contrast, electrically stimulated cells exhibited a more elongated morphology and greater interaction (Figure 4a). Quantitative analysis of the length‐width ratio, a key indicator of cardiac maturation,^7^ showed a significant increase in both electrically stimulated groups, confirming that ES promotes cardiomyocyte elongation (Figure 4b). Additionally, perfusion had a significant effect on sarcomere length, showing a statistically significant increase compared to the non‐stimulated group and both static groups (Figure 4c). In summary, electrical stimulation plays a key role in promoting cardiomyocyte elongation and influencing sarcomere organization.

### 2.4 Gene expression of cardiac biomarkers

The impact of perfusion and its combination with ES was investigated by analyzing the transcriptional analysis of cardiac genes related to the contractile unit: myosin heavy chain‐α (*Myh6*) and ‐β (*Myh7*), myosin light chain‐2 (*Myl2*) and ‐7 (*Myl7*) (Figure S4) and troponin I3 (*Tnni3*) as well as genes involved in electrical conduction, gap junction protein αlpha‐1 (*Gja1*) and calcium binding protein calsequestrin 2 (*Casq2*). The ratios *Myl2*/*Myl7* and *Myh6*/*Myh7* were used as indicators for the switch from fetal‐like phenotype characterized by the expression of *Myh7* and *Myl7* to adult rat cardiomyocyte phenotype *Myh6* and *Myl2*.^7^ The *Myl2*/*Myl7* ratio was found to be highest in the perfusion − ES group, despite high intergroup variability. However, these differences were not statistically significant (Figure 4d). The ratio *Myh6*/*Myh7* was higher in both perfusion cultures compared to static conditions, with the highest levels observed in the perfusion − ES group (Figure 4e). These ratios mirrored the *Myl2*/*Myl7*, indicating a switch to a mature cardiomyocyte phenotype. Interestingly, the gene expression related to electrical conduction, *Gja1* and *Casq2*, was the highest in the perfusion − ES, albeit all results being non‐significant, while the expression of *Tnni3* did not change across all groups (Figure 4f).

### 2.5 Construct remodeling

**FIGURE 5:**
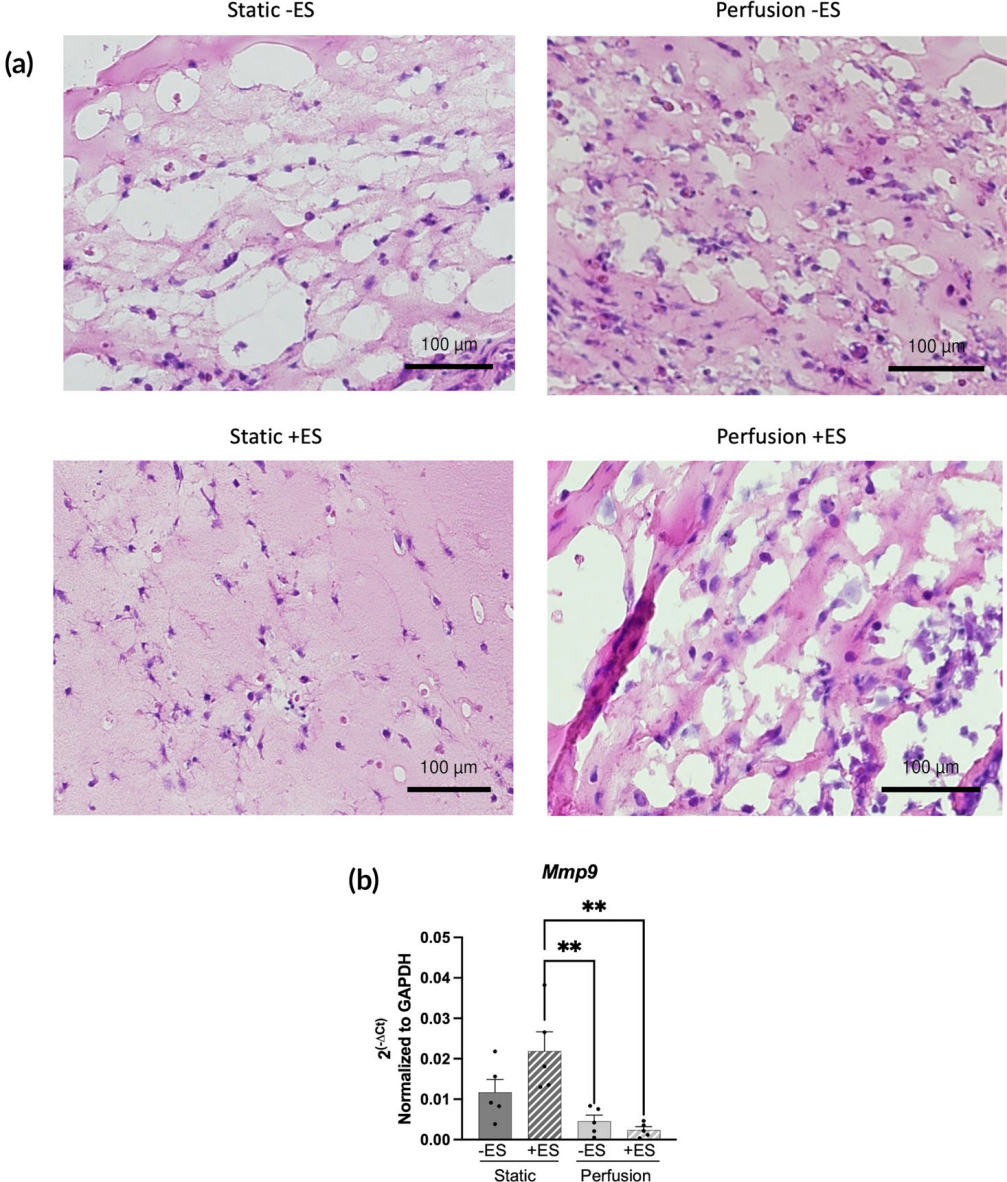
Construct remodeling. (a) Representative H&E images for construct generated either in static or perfusion condition with or without electrical stimulation (±/− ES). (b) Gene expression in RT‐PCR for ECM remodeling gene *Mmp9*. Comparisons were performed using a two‐way ANOVA and normalized to *Gapdh* as a housekeeping gene. All data are represented as mean ± SEM. (**p < 0.01). RT‐PCR analysis was performed on a minimum of four samples from a minimum of two independent experiments.

Cardiac constructs cultured under perfusion exhibited denser extracellular matrix (ECM) deposition, whereas those cultured under static conditions showed higher porosity and more isolated cells. Representative H&E staining demonstrated a higher overall presence of cells, in accordance with cell quantification in Figure 3a and a higher construct remodeling in both perfusion groups, more pronounced in the perfusion + ES (Figure 5a). The restructuring of the ECM suggests superior tissue remodeling in perfusion groups, particularly under electrical stimulation conditions. To further investigate construct remodeling, the transcriptional expression of genes involved in extracellular remodeling was analyzed: matrix metallopeptidase‐9 (*Mmp9*). *Mmp9* expression was 4‐fold higher in the static + ES group compared to both perfusion groups, with statistical significance and twice‐fold compared to the static − ES group. Overall, these results indicate that perfusion, especially in combination with ES, supports superior tissue remodeling by promoting ECM–protein turnover.

### 2.6 Construct functionality

**FIGURE 6:**
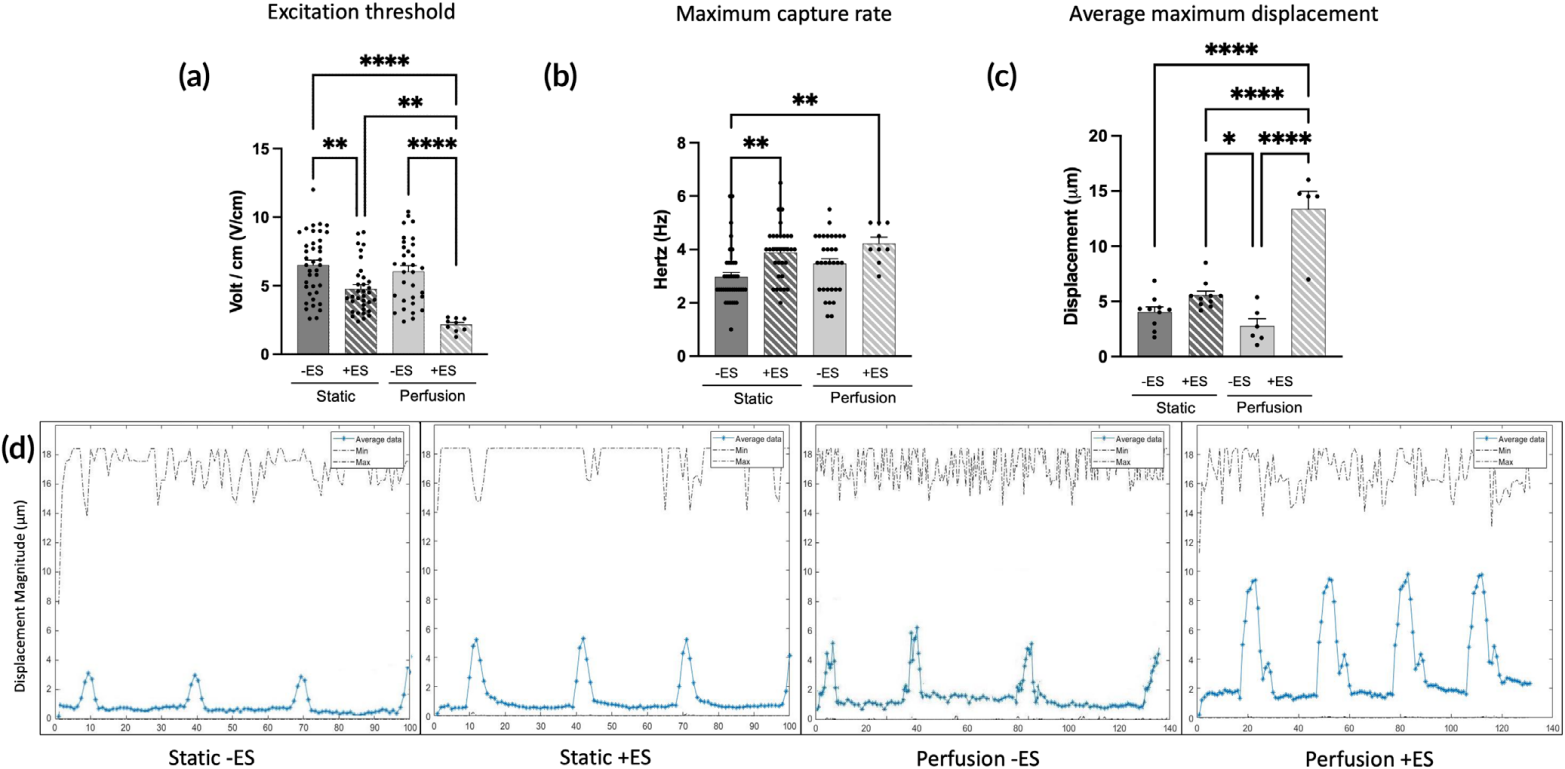
Construct functionality. Graphs displaying (a) excitation threshold (ET), (b) maximum capture rate (MCR) comparisons, and (c) average pixel displacement (APD). (d) Displacement plot over multiple contractions during MCR measurements. Comparisons were performed using a two‐way ANOVA and a Tukey's post‐hoc test. (*p < 0.05; **p < 0.01; ****p < 0.0001). Functionality testing was performed on a minimum of four samples from a minimum of two independent experiments.

Live‐cell video analysis under external pacing revealed that the excitation threshold (ET) was significantly lowest in the perfusion + ES group (2.2 ± 0.16 V/cm). In contrast, all other groups were found to have a significantly higher ET, static − ES (6.5 ± 0.37 V/cm) and static + ES (4.8 ± 0.31 V/cm), as well as the perfusion − ES group (6.0 ± 0.42 V/cm). Noteworthy, there was no significant difference between the perfusion − ES and the static − ES groups (Figure 6a). ES significantly reduced ET, compared to the corresponding non‐stimulated groups.

The maximum capture rate (MCR) was lower in the non‐electrically stimulated groups, static − ES (3.0 ± 0.17 Hz) and perfusion − ES (3.5 ± 0.19 Hz), compared to the electrically stimulated groups: static + ES (3.9 ± 0.18 Hz) and perfusion + ES (4.2 ± 0.24 Hz). Electrical stimulation significantly increased MCR compared to the non‐stimulated Static group (Figure 6b).

Extended image analysis measured pixel displacement per contraction, revealing a similar trend. The perfusion + ES groups (6.5 ± 1.2 μm) showed a greater average pixel displacement (APD) compared to the non‐stimulated groups (static − ES: 2.1 ± 0.19 μm, perfusion − ES: 1.6 ± 0.31 μm) (Figure S5). Notably, the perfusion − ES group also showed significantly lower displacement than the electrically stimulated non‐perfusion group (Static + ES: 3.4 ± 0.27 μm).

The average maximum displacement per contraction of individual pixels (Figure 6c) followed the same pattern, with the perfusion + ES group displaying a significantly higher value. Representative contraction analyses, including a contraction magnitude at different contraction stages contraction (Figure 6d) and vector displacement maps for a perfusion + ES construct (Figure S5) illustrate these findings across all constructs. A summary of the findings can be found in Table S2.

## 3 DISCUSSION

This study presents and investigates a bioreactor that combines direct perfusion‐based culture with electrical stimulation to improve the maturation of cardiomyocyte tissue within a fibrin‐based hydrogel. Direct perfusion enables the maintenance of viable tissue thicknesses exceeding the physiological oxygen diffusion limit (approximately 100–300 μm), as demonstrated in 2 mm‐thick constructs. Electrical stimulation provides an additional biomimetic cue that can further promote cardiac cell maturation. Together, these approaches are promising as a potential solution to key limitations in the engineering of thick, functional cardiac tissues.

Many bioreactor designs are proprietary and custom‐made in a complex fashion, often requiring in‐depth training on usage. One of the main aims of this study is to present a simple, easily modifiable bioreactor, designed to be integrated with an already existing and well‐established setup. The bioreactor system is a culture setup that has previously been used to investigate different cell types.^40^, ^41^, ^42^ A 3D printed mold was used to create a custom PDMS insert at a minimum financial cost, in which modifications could be rapidly applied. Staubli *et al*.^26^ developed a method to directly perfuse a soft hydrogel by punching holes using equally spaced cannulas. We adapted this method for use with fibrin‐based hydrogels,^43^ primarily as fibrinogen is among the most used scaffolds in cardiac tissue engineering and is promising in terms of compatibility,^31^ extended culture time,^44^ and scalability.^12^, ^45^

In contrast to indirect perfusion, direct interstitial perfusion improves the distribution of nutrients and oxygen. Furthermore, it efficiently removes waste products and prevents the local accumulation of electrochemical byproducts generated during electrical stimulation. The viable tissue thickness can be increased while maintaining the tissue ultrastructure^12^ mimicking *in vivo* circulation circumstances. The generation of constructs of clinically relevant size^46^ is imperative for the translation of engineered tissues into living hosts, not only humans. We demonstrate that functional tissues of 2 mm thickness and 8 mm diameter can be reproducibly created. The theoretical scalability of the proposed bioreactor set‐up is supported using constant medium perfusion and uniform distribution of the channels. Moreover, the perfusion flow is primarily intraluminal through the channels. Due to the low pressure drop and the soft nature of the fibrin, forced interstitial flow is negligible. Instead, the channels act to shorten the diffusion distance, facilitating mass transport into the gel bulk similar to a capillary network without subjecting the cells to high interstitial shear stress.

At the time of seeding, cell density and composition were comparable across all experimental groups. However, after 7 days of culture, the perfusion group exhibited twice the number of DAPI^+^ cells (Figure 3a), suggesting a higher total cell count. Differences in functional outcomes may be attributed to the greater number of viable cells maintained under perfusion. This observation is consistent with previous studies, which have shown that perfusion culture supports, and in some cases increases, cell viability and proliferation.^12^, ^47^ Interestingly, the cellular composition of the final gels varies greatly as quantified in the immunofluorescence analysis. Perfusion significantly enhanced the number of cardiomyocytes, particularly those exhibiting higher levels of cardiac differentiation, especially in the presence of ES (Figures 3d and S3). This may be explained by the limited nutrient diffusion in static cultures, which likely leads to selective cell death of metabolically demanding cells such as cardiomyocytes. Beyond individual markers, the observed gene expression patterns suggest that the bioreactor modulates tissue phenotype through two distinct but synergistic mechanisms. First, the data suggest that perfusion primarily acts by mitigating hypoxic and inflammatory signaling rather than through direct mechanical forces. Static culture was characterized by high *Mmp9* expression (Figure 5b), a metalloproteinase often upregulated by stress‐responsive pathways such as but not limited to NF‐κB and HIF‐1α,^48^ in response to nutrient limitation. By ensuring stable biochemical gradients, perfusion significantly reduced *Mmp9*. Second, the addition of electrical stimulation appears to promote calcium‐dependent maturation processes, as reflected by improved functional performance of the constructs.

Interestingly, both static groups and the perfusion group subjected to ES showed a remarked reduction in the number of activated fibroblasts (Figure 3e). This suggests that perfusion alone may induce a phenotype switch in cardiac fibroblasts toward a myofibroblast state, a transition that appears to be mitigated by the presence of ES. The influence of direct perfusion pressure,^49^, ^50^ as can be estimated when using a porous ECM scaffold^35^ does not apply in the case of our proposed bioreactor. As the cells are directly encased in the fibrin structure, the culture medium passes through the preformed channels (Figure 2b). Nevertheless, it may still be relevant during the late phase of culture, after significant hydrogel degradation or tissue remodeling has occurred. Due to the soft nature and remodeling effect of the cells,^51^ the hydrogel can be modified by the cellular orientation by cues such as strain^18^ or substrate micropatterning,^52^ to name a few. Protein and matrix deposition may lead to a reduction in pore size, thus increasing fluid resistance. A bell shape would arise, inflicting radial stretch toward the outside edge of the construct. This was not observed in our bioreactor set‐up, probably due to the thin PDMS membrane the hydrogel was seeded on, acting as stiff resistance. Collectively, these findings indicate that perfusion acts primarily by mitigating metabolic stress and establishing a stable biochemical baseline, rather than through direct mechanical mechanotransduction, thereby creating the permissive environment necessary for cellular maturation.

By day 7, active remodeling likely alters the mechanical environment, at which point perfusion may contribute to both cardiomyocyte dedifferentiation and fibroblast activation.^53^

The combination of multiple cues, especially electrical stimulation, has long been recognized as a critical factor in cardiac tissue engineering.^10^ Numerous bioreactor designs have been developed, many yielding promising results; however, their scalability often remains limited.^30^, ^54^ This limitation may also be in part due to the high estimated cost per construct. Excluding cell sourcing and general laboratory expenses, the complexity of many bioreactor systems contributes significantly to cost and restricts widespread use.^55^ In contrast, the bioreactor presented in this study leverages a simplified and cost‐effective design using PDMS and 3D printing of the reusable molds, allowing for rapid and flexible modifications. The system is also adaptable in hydrogel size, both in height and diameter. Notably, an upscaled version of the configuration has previously been used to produce 50 mm diameter patches.^46^, ^56^ In theory, the construct length could be extended to reach the entire fluid culture chamber of the PDMS insert. A limitation of certain designs is the inability to perform real‐time monitoring of the constructs. Although PDMS is in fact a see‐through polymer, the configuration did not support visual access in this setup. Nevertheless, the system can be readily modified to incorporate monitoring features, as demonstrated by previous groups,^17^, ^35^ which included gold electrodes for real‐time electrocardiogram‐like signal recordings. This approach further bridges the translational gap of monitoring engineered tissues in a way that can be applied in vivo.

Electrical stimulation has been extensively investigated with numerous protocols and comprehensive reviews are available.^57^ One method involves gradually increasing the stimulation frequency by 0.33 Hz daily, reaching up to 6 Hz over an extended culture period.^58^ A short recovery period following cell seeding is commonly adopted^59^ to minimize cell stress and allow for initial organization. In our experimental plan, ES was started on day 3 and maintained at fixed parameters throughout the culture period of an additional 4 days. Such a relatively short culture period did not allow for progressive adjustment of electrical parameters. However, longer culture periods have been shown to promote cardiomyocyte maturation.^60^ Despite the limited culture period, our study revealed significant functional improvements.

The perfusion + ES group demonstrated the highest average contraction displacement (Figure 6c), as quantified by video analysis, likely reflecting its superior cellular composition and further underlining once again the importance of electrical stimulation on cardiomyocyte culture. This trend was further confirmed by improvements in the ET (Figure 6a), as well as MCR (Figure 6b), both key indicators of electrical responsiveness and maturity. At the cellular level, the average sarcomere length was significantly greater in both electrically stimulated groups (Figure 4c), although values remained below those reported for adult cardiomyocytes.^7^, ^61^ This discrepancy likely reflects the relatively short culture time and the absence of active relaxation before cell fixation, a factor that could introduce systematic underestimation across groups. Despite this limitation, the observed intergroup differences in sarcomere length remain informative.

The average cell length and width aligned with the physiological ranges previously published. Neonatal cardiomyocytes are reported to have a length of 21.7–44.7 μm and a width of 7.5–8.5 μm, whereas adult rodent cardiomyocytes reach lengths of 100–128 μm and widths of 20–25 μm.^62^ The observed increase in length‐to‐width ratio further supports the trend toward cardiomyocyte maturation, which evolves from 2.9 in neonatal cardiomyocytes to 5.3 in adult cells (Figure 4b,c).^7^

These findings reinforce the role of ES in promoting cellular maturation from an early time point. Moreover, the type of waveform used for ES^38^, ^63^ may represent an additional optimization parameter worth exploring within the current bioreactor system to further enhance maturation outcomes.

Further gene expression analysis did not reveal statistically significant differences among groups in key markers for contractile and electrical units. There was, however, a trend indicating a switch from fetal to mature isoforms of myosin heavy chains for all groups. (Figure S4). To further investigate this phenotype switch, the ratios of the myosin light chains (*Myl2/Myl7*) and the heavy chains (*Myh6/Myh7*) were calculated (Figure 4d,e). For the light chains, the perfusion − ES group showed a non‐significant increase of nearly 100% compared to the other groups. For the heavy chains, both perfusion groups demonstrated a trend toward mature gene expression compared to the non‐perfusion groups. Interestingly, this does not coincide with the findings by Visone *et al*.^35^ who investigated a bioreactor combing the interstitial perfusion of porous scaffolds combined with ES. In their analysis of the cardiac heavy chains, no significant increase in the mature phenotype was found. The intervention group of bidirectional perfusion combined with ES even led to an increased ratio of fetal to mature gene expression.

ECM remodeling, an essential component supporting cardiac tissue maturation, was also assessed through the expression of the metalloproteinase *Mmp9* (Figure 5b). *Mmp9* expression is higher in both static culture groups. Since *Mmp9* degrades type IV collagen and is a major inflammatory mediator,^64^ its elevated expression aligns with the higher fibroblast populations and denser ECM in these groups (Figures 3e and 5a).

This might again be attributed to varying cellular compositions of the engineered tissues, with the static groups including a higher relative number of fibroblasts in the construct population (Figure 3b). The favorable effect of perfusion likely originates from its capacity to maintain cardiomyocyte populations, whereas ES appears to specifically enhance their maturation. The combined application of perfusion and ES yields superior functional outcomes, although the mechanisms underlying their synergy remain to be fully understood.

## 4 MATERIALS AND METHODS

### 4.1 Ethics statement

Neonatal rat cardiomyocytes used in this study were isolated from 2/3‐day old Sprague–Dawley rat pups of unknown sex. All animals were kept and euthanized according to Swiss Federal guidelines for animal welfare; all procedures were approved by the Veterinary Office of the Canton Basel, Switzerland (Approval No: 2608_30631).

All materials were purchased from Sigma‐Aldrich (Merck Millipore, MI, USA) and the reference number is given unless otherwise stated.

### 4.2 Bioreactor design

#### 4.2.1 Custom perfusion‐bioreactor inset

A 3D‐printed mold (Figure 1a) was designed using Solidworks (Dassault systèmes, MA, USA) and printed using a Prusa i3 3D printer (Prusa research a.s., Prag, CZ). It was designed to snugly fit the lid of a U‐tube bioreactor.^65^ An analogous system is commercially available (Cellec Biotek AG., Basel, CH). An aluminum cylinder of 10 mm outer diameter was placed centrally to retain the internal fluid canal. Using a 25G cannula (B. Braun, Melsungen, DE) a perpendicular hole was drilled 3 mm from one end of a 32 mm long, 3 mm diameter graphite rod (Ref. 496,537). Subsequently, a 6 cm long platinum wire of 0.3 mm diameter (Faust Laborbedarf AG., Schaffhausen, CH) was introduced into the hole and wound around the rod, so 5 cm of wire would remain free. A pair of these graphite rods were placed horizontally and with opposite ends into the mold and onto the internal lip of the lid, so to be resting against the sides of the aluminum cylinder, 10 mm apart. This equals the working distance between the electrodes. The free platinum wire ends of both carbon rods were fed through a respective 0.3 mm hole in the mold sidewall. Screws were held in place by lab tape and pushed through the screw holes of the lid to create the screw canals. The mold was then filled with a centrifuged (60 s, 3000*g*) 2‐component PDMS silicone Elastomer (Sylgard 184, Dow Europe, Horgen, CH) at a ratio of 1:10 and degassed in multiple steps (3 min, ×) in a vacuum chamber for a total of 15 min. After a 24 h curing period at 60°C, the mold and aluminum insert were removed and the PDMS insert left to fully cure for 7 days at room temperature. The internal fluid canal was checked for silicone residues, and the graphite rods were cleaned, so to be fully exposed to the cell culture media throughout the width of the canal. The bottom chamber was attached and fixed by screws to test for water tightness.

A 1 mm thick PDMS membrane was made by pouring the equivalent amount measured by weight of elastomer into the lid of a 60 mm petri dish, degassed and cured as described above. Afterwards, 12 mm discs were punched. The resulting discs were checked for consistent thickness with a caliper and left to fully cure for 7 days at room temperature. All parts were subsequently sterilized using a standard steam autoclave. A total volume of 8 mL of culture medium was used in each perfusion bioreactor.

#### 4.2.2 Custom static‐culture inset

A 3D printed negative‐well inset was designed to fit centrally in each well of a standard 6 well‐plate.^38^ A standard six‐well plate containing 8 mL of culture medium was used as the static control, as it provides the same total medium volume as the perfusion system while avoiding confinement‐related diffusion limitations inherent to a no‐flow condition within the bioreactor chamber. PDMS was poured around the central inset, so as to be 20 mm high, degassed and cured as described above. After removing the central inset, a pair of graphite rods with platinum wires attached were placed in the preformed grooves 10 mm apart, analog to the perfusion‐bioreactor inset. The free ends of platinum wires were long enough to close the lid of the well‐plate and still retain 2 cm of wire. All parts were subsequently sterilized using a standard steam autoclave. The distance between the two carbon electrodes is 10 mm. 3D‐Models can be found in Appendices S1 and S2.

#### 4.2.3 Computational fluid dynamics simulations

To investigate the development of the culture medium flow inside the culture chamber and the fluid flow‐induced shear stress on the hydrogel construct, computational fluid dynamics simulations were performed using the commercial finite‐element simulation software Comsol Multiphysics 6.0 (Comsol Inc., Stockholm, SWE) (Figure 1b,c). The 3D geometries of the culture chamber and of the hydrogel construct with perfusion channels were imported and the resulting fluid domain was discretized with 1.48 × 10^7^ total elements, using tetrahedral elements for the bulk and hexahedral elements for the boundary layers. The culture medium was modeled as an incompressible, Newtonian fluid (density *ρ* = 9.94 × 10^2^ kg/m^3^, dynamic viscosity *μ* = 6.89 × 10^−4^ Pa s at 37°C). A steady‐state simulation was performed prescribing uni‐directional perfusion (flow rate *Q* = 0.3 mL/min), imposing a parabolic velocity profile at the inlet of the culture chamber. A reference pressure was imposed at the outlet, and the no‐slip condition was applied at the internal walls of the culture chamber and at the external surface of the hydrogel construct. The simulated flow regime within the culture chamber, following the evaluation of Reynolds number (*Re*) calculated considering the internal diameter of the inlet channel (3.5 mm) as the characteristic length, resulted to be laminar (*Re* = 2.62). Consequently, the incompressible form of the Navier–Stokes and continuity equations in their discretized form were solved.

#### 4.2.4 Electric field finite element analysis

For characterizing the spatial distribution of the electric field and of the current density within the construct, a finite element analysis was performed (Comsol Multiphysics 6.0, Comsol Inc.) (Figure 1d,e). The geometry of the culture chamber, composed of five sub‐domains (PDMS inset; carbon rod electrodes; culture medium; polycarbonate bioreactor structure; hydrogel construct), was meshed with 2.73 × 10^6^ tetrahedral elements and 1.6 × 10^5^ triangular elements. Each sub‐domain was assumed as a homogeneous isotropic medium with specific electrical properties (Table S1). A stationary simulation was performed, solving the continuity equation in absence of distributed current sources:

$$\nabla \cdot J=\nabla \cdot \left(\mathrm{\sigma E}+J_{e}\right)=0$$

where *J* is the current density (A/m^2^), *σ* is the electrical conductivity (S/m), *E* is the electric field distribution (V/m), and *J*_*e*_ is the externally generated current density (A/m^2^). *J*_*e*_ was set to 0 in the simulation and *E* was derived as the gradient of the electric potential *V*:

E=−∇V

As boundary conditions, the external surfaces of the electrodes were set at a uniform electric potential (*V* = 3.5 or 6.08 V for the positive electrode, *V* = 0 V for the ground electrode) and electric insulation was imposed at the external surfaces of the model.

### 4.3 Cell isolation

Neonatal rat cardiomyocytes were isolated according to a previously described protocol.^12^ In short, 2/3‐day old Sprague–Dawley pups were euthanized and the hearts collected. After removing major vessels and atria, the ventricles were minced into equal pieces and digested overnight in 0.06% w/v trypsin (Ref T1426) in HBSS (Ref H6648). The next day, the tissues were transferred into a 500 mL flask and digested in 0.125% (w/v) type II collagenase (Worthington Biochemical Corporation, Lakewood, USA) for 4 min at 37°C and shaking at 70 rpm. The digestion was repeated a total of five rounds. Isolated cells were plated for 45 min in a polystyrene culture flask in high‐glucose cell culture media. After this pre‐plating step, the non‐adherent cardiomyocytes were used immediately in the experiments. All subsequent cell culture was performed using low‐glucose cell culture media. Cell culture media: 88% (v/v) DMEM High glucose (Ref. D6429) or DMEM Low glucose (Ref. D6046), 10% FBS (Ref. F9665), 1% v/v HEPES solution (Ref. H3537) and 1% (v/v) Penicillin–Streptomycin (Ref. P4333). Tranexamic acid (Ref. 857653) was added at 160 μg/mL, as an alternative to Aprotinin to counteract fibrinolysis.^66^

### 4.4 Hydrogel fabrication

A fibrin hydrogel was chosen as an easily modifiable, component‐based mixture. Neonatal rat cardiomyocytes were used immediately after isolation. A total hydrogel volume of 100 μL was cast onto a PDMS membrane, creating a cylinder of 8 mm diameter and 2 mm height.^26^ For both static and perfusion groups, the cell seeding protocol is identical and all concentrations given are final volume concentrations. Human thrombin (Ref. T6884) at 5 U/mL was mixed with CaCl_2_ (Ref. C4901) at 4 mM/mL and Tranexamic acid dissolved in cell culture media at 400 μg/mL. The isolated cardiomyocytes were added at 10 million/mL and resuspended. Human fibrinogen (Ref. F3879) at 20 mg/mL was added slowly and the total mixture, measuring 100 μL, was transferred immediately onto the center of the PDMS disk. The hydrogel was left to cure covered for 5 min, then placed in a standard incubator at 37°C, 97% humidity and 5% CO_2_ for a further 25 min. Subsequently, a custom hole punch was used as previously described,^26^ to add 0.5 mm perfusion channels (Figure S1), and the PDMS disc is placed either on the lid or in the center of a 6‐well‐plate. The final concentrations of fibrinogen (20 mg/mL) and thrombin (5 U/mL) allowed for an estimated structural modulus of around 1.5 kPa.^67^ CaCl_2_ was added as a catalyst for the proteolysis of fibrinogen by thrombin (Figure 1a).

### 4.5 Cell seeding and culture

For the static groups, design features allowed a fit into a standard 6‐well plate. The PDMS well inserts were placed around the hydrogel and 8 mL of cell culture media was filled.

For the perfusion groups, the hydrogel was placed on the lid of the bioreactor, the custom PDMS perfusion‐bioreactor inset placed on top, and the bottom fixed with screws. Media flow was initiated using a peristaltic pump (Ismatec, IDEX Health & Science LLC, Middelborough, USA) at a flow rate of 0.3 mL/min. Using a custom stimulation module, in the +ES groups, electrical stimulation was initiated by connecting the stimulation module to the protruding platinum wires on day 3. Monophasic square waveforms were applied with a frequency of 1 Hz, pulse duration of 2 ms, and amplitude of 3.5 V for static and 6.08 V for perfusion (Figure S1) conditions, corresponding to an electrical field of 3 V/cm based on computational analysis. Parameters were selected based on established protocols to ensure physiological pacing while minimizing cell damage.^10^, ^17^, ^19^, ^30^, ^35^, ^68^ Media change occurred every 2 days until day 7. Hydrogel components and volumes are listed above (Figure 1a). A total of four experimental groups were compared: static without electrical stimulation (Static − ES), static with electrical stimulation (Static + ES), perfusion without electrical stimulation (Perfusion − ES), and perfusion with electrical stimulation (Perfusion + ES) (Figure 1b). Photographic representation of the bioreactor set‐up is shown in Figure S2.

### 4.6 Analysis

#### 4.6.1 Functional testing

All cell constructs were removed from their respective culture chamber, placed in fresh prewarmed cell culture media and transferred to the live‐cell microscope chamber (prewarmed at 37°C, 95% humidity, 5% CO_2_) of an Olympus BX63 (Olympus, Shinjuku, JP). A new static PDMS insert was placed in a 6‐well plate and the platinum wires were connected to the custom stimulation module. Electrical stimulation (1 Hz and 2 ms) was commenced under observation at 2× and 4× magnification and the amplitude was increased stepwise until the whole construct contracted simultaneously (Videos [Link], [Link]). This voltage level relates to the excitation threshold (ET). The voltage was set at 1.5× the ET and then the frequency was increased stepwise until the hydrogel contractions no longer followed the pacing frequency. This maximum frequency before failure relates to the maximum capture rate (MCR). After test completion, hydrogels were either fixed in 4% paraformaldehyde (PFA) (Ref. 158127) at 4°C overnight for subsequent histology or flash frozen unfixed at −80°C for quantitative real‐time PCR.

#### 4.6.2 Quantitative real‐time RT‐PCR

After thawing, constructs were cut into pieces and homogenized at 15,000 rpm in TRIzol and RNA was extracted using a TRIzol extraction kit (Zymo Research, Irvine, USA). After RNA quantification and respective dilution using a nanodrop spectrophotometer (Thermofisher Scientific, Waltham, USA) and nuclease‐free water (Ref. W4502), random primer (Ref. C118A, Promega, Madison, USA) was added. After thermocycling (Automated thermal cycler, Thermofisher Scientific) for 10 min at 70°C, reverse transcriptase mix consisting of superscript III reverse transcriptase (Ref. 56575, Invitrogen), solution of deoxynucleotide triphosphates (Ref. U151B, Promega), 5× first strand buffer (Ref. Y02321, Invitrogen), and dithiothreitol (Ref. y00147, Invitrogen) was added and the samples were thermocycled for a further 10 min at 25°C, 30 min at 48°C, and 10 min at 95°C.

cDNA samples were then analyzed for the individual genes diluted in TaqMan assays master mix (Ref. 4369016, Thermofisher Scientific) using an ABI 7300 RT‐PCR cycler (Applied Biosystems, Waltham, USA).

All genes used were purchased from Thermofisher Scientific: Connexin43 (*Gja1*, Rn01433957_m1), Troponin‐I (*Tnni3*, Rn00437164_m1), Myosin light chain 2 ventricular (*Myl2*, Rn02769676_s1), Myosin light chain 7 atrial (*Myl7*, Rn01752521_g1), Myosin heavy chain 7 (*Myh7*, Rn01488777_g1), Myosin heavy chain 6 (*Myh6*, Rn00691721_g1), Matrix metallopeptidase 9 (*Mmp9*, Rn00579162_m1) and Calsequestrin 2 (*Casq2*, Rn00567508_m1). All gene expression was normalized to glyceraldehyde‐3‐phosphate dehydrogenase (*Gapdh*, Rn01775763_g1).

#### 4.6.3 Video analysis

Functional testing videos underwent segmentation and post‐processing using a custom matlab script (Mathworks, Portola Valley, USA) as described previously.^18^ Briefly, digital image correlation (DIC) was implemented to assess the average pixel displacement (APD) (Videos  and S2), contraction vector field (Video S3), and the strain.

#### 4.6.4 Histological analysis

After overnight fixation in 4% PFA, hydrogels were rinsed in 1× PBS and frozen in optimal cutting temperature compound embedding matrix (Cellpath Ltd., Powys, UK). 10 μm sections were cut using a conventional cryostat (Leica Biosystems, Wetzlar, DE) and stained according to standard protocols for Hematoxylin and Eosin (H&E) or immunofluorescence. In detail, slides were permeated with 0.2% Triton‐X (Ref. 93443) for 10 min, followed by a blocking for 1 h with 5% bovine serum albumin (BSA) blocking solution (Ref. A9576). The following primary antibodies were incubated overnight at 4°C: anti‐sarcomeric α‐actinin (SAA, ab9465, and Abcam), anti‐cardiac troponin T (cTNTab8295, Abcam), anti‐α‐smooth muscle actin (α‐SMA, Ref. A2547), anti‐Ki67 (ab15580, Abcam). After three washes with PBS, the following secondary antibodies were left to incubate for 1 h at 1:200 in 1% BSA blocking solution: Alexa 488 (A21141 and A11008, Life Technologies, Carlsbad, USA), Alexa 546 (A21123, Life Technologies), Alexa 647 (A21245 and A21241, Life Technologies). During this incubation, 4', 6‐diamidino‐2‐phenylindole (DAPI, Ref. D9542) was also added for nuclear staining. All primary antibodies were diluted and used according to the average recommended concentration in 1% BSA blocking solution. Coverslips were applied using DAKO mounting medium (Agilent, Santa Clara, USA), and the slides were stored at 4°C.

#### 4.6.5 Image analysis

Immunofluorescence images were acquired using a widefield‐fluorescence Nikon Ti2 inverted microscope (Nikon, Minato, Japan) at different magnifications. Subsequent image processing and analysis were performed using Fiji (ImageJ, NIH, Public Domain) and QuPath v.0.5.1.^69^ In detail, images were cropped to remove blank areas and loaded into the QuPath project. For the average sarcomere length analysis and the cell length to width ratio, a minimum of eight cells and 60 sarcomeres from each condition were analyzed. Upon demand, the script can be made available.

#### 4.6.6 Statistical analysis

All statistical analysis was performed using Prism 10 (Graphpad, Boston, USA) and statistical significance was determined to be at *p < 0.05 and further denominated with stars (**p < 0.01, ***p < 0.001, and ****p < 0.0001). All data are presented as standard errors of the mean (SEM) unless otherwise stated. Data sets were assessed for normal distribution using the Kolmogorov–Smirnov test. If normally distributed, data were analyzed using a two‐tailed unpaired Student's *t* test or analysis of variance one‐way ANOVA for single comparisons and multiple comparisons respectively, followed by Dunn's or Tukey's post‐hoc. Data deemed not normally distributed were analyzed using a non‐parametric Mann–Whitney test or Kruskal–Wallis test for single comparisons and multiple comparisons respectively. For data analyzed by means of a two‐way ANOVA, a Tukey's post‐hoc test was used. All histological and immunofluorescent analyses were performed on a minimum of two independent experiments with a minimum of two replicates. PCR analysis was performed on a minimum of four samples from a minimum of two independent experiments. Functionality testing was performed on a minimum of four samples from a minimum of two independent experiments.

## 5 CONCLUSION

In summary, we propose an easy yet effective adaptation of a commercially available bioreactor to enable direct perfusion of soft hydrogels in combination with electrical stimulation. This proof‐of‐concept study demonstrates that the integration of these two stimuli significantly enhances the structural and functional maturation of engineered cardiac tissues. While the results are promising, further investigation is required to validate its scalability and applicability to generating clinically relevant tissue constructs of greater size and complexity. Designed to be open‐source, cost‐efficient, and easily customizable, this bioreactor provides a versatile and accessible platform not only for cardiac tissue engineering but also for the development of other soft tissue models requiring control and combined application of multiple biophysical cues.

## AUTHOR CONTRIBUTIONS

G.R.: Project design, data acquisition, data editing and formatting, manuscript writing. S.G.: Data acquisition and manuscript writing. A.S.: 3D printing, mechanical/Engineering expertise, data acquisition. D.F.: Data acquisition, microscope expertise. G.I.: Project design, troubleshooting, model establishment. D.M.: Collaboration and project management, data revision. G.M.: Manuscript revision, data acquisition, project management. A.M.: Total Project management and supervision. All authors reviewed the manuscript.

## FUNDING INFORMATION

This work was supported by a Swiss National Science Foundation grant (310030_1789).

## CONFLICT OF INTEREST STATEMENT

The authors declare no conflicts of interest.

## Supporting information


**APPENDIX S1:** 3D‐software rendering of the complete bioreactor set‐up (explosion diagram).

**APPENDIX S2:** 3D‐software rendering of the complete bioreactor set‐up (non‐explosion diagram).

**FIGURE S1:** Bioreactor design. (a) 3D model of the perfusion bioreactor. The hydrogel is in pink with visible channels. (b) Schematic representation of the bioreactor chamber set‐up. (c) Line graph of the velocity magnitude. (d) Current density simulation throughout the tissue construct horizontal plane. (e) Close up representation of wall shear stress inside the perfusion channels, in mPa. Arrow signals directions of fluid flow. (f) Close up representation of the channel perfusion velocity in mm/s. Arrow signals directions of fluid flow.

**FIGURE S2:** Bioreactor set‐up. (a) Bioreactor assembly. (b) Channel punch. (c) PDMS well insert (static). (d) PDMS well insert, in position in a six‐well plate. The (+) and (−) indicate the electrical stimulation connection to the platinum wires. (e) PDMS bioreactor insert (perfusion). (f) PDMS disk in position with hydrogel atop. (g) PDMS bioreactor in position atop the bioreactor chamber lid. (h) Full mount with screws, side view. Arrow indicates media flow direction. (j) Bird's eye view inside the perfusion chamber with visible hydrogel. (k) Full perfusion set‐up with media‐reservoir and tubing. (l) Assembled perfusion bioreactor. The (+) and (−) indicate the electrical stimulation connection to the platinum wires. Arrows indicate media flow direction. The circles indicate positioning of roller pump.

**FIGURE S3:** Immunofluorescent analysis. (a) Immunofluorescent staining for DAPI in blue and Ki67 in green. (b) Immunofluorescent staining for DAPI in blue, cTNT in red and aSMA in green. (c) Quantification of cTNT^+^ and DAPI^+^ co‐localized cells. Data are represented as mean ± SEM. Comparisons were performed using a two‐way ANOVA and a Tukey's post‐hoc test (*p < 0.05, **p < 0.01, ****p < 0.0001).

**FIGURE S4:** Myosin chain analysis. Gene expression in RT‐PCR of relative increase of the myosin chain units: *Myl2, Myl7, Myh6, Myh7*. Comparisons were performed using a two‐way ANOVA test and normalized to *Gapdh* as a housekeeping gene. All data are represented as mean ± SEM. RT‐PCR analysis was performed on a minimum of four samples from a minimum of two independent experiments.

**FIGURE S5:** Average pixel displacement. (a) Average pixel displacement of minimum and maximum displacement combined. Comparisons were performed using a two‐way ANOVA and a Tukey's post‐hoc test. (b) Vector map example for a perfusion + ES construct read‐out. All data are represented as mean ± SEM (*p < 0.05, ***p < 0.001, ****p < 0.0001).

**TABLE S1:** List of electrical conductivity (S/mm) and relative permittivity of materials used. References are given.

**TABLE S2:** Summary of the stimulation parameters, flow settings, and key findings for each experimental group.

**VIDEO S1:** Example perfusion + ES construct. Example video of microscope image (2×) taken at 33 fps, of a perfusion + ES construct spontaneously beating with close‐up cut out of point–point analysis.

**VIDEO S2:** Contour plot of displacement magnitude. Example video showing a contour plot of the displacement magnitude of a perfusion + ES construct.

**VIDEO S3:** Vector field plot of displacement. Example video showing a vector field plot of the displacement of a perfusion + ES construct.

## Data Availability

The data that support the findings of this study are available from the corresponding author upon reasonable request.
