# Supplementary figures and images for: Open‐source bioreactor delivers electrical and perfusion stimulation supporting 3D cardiac engineered tissue maturation
Source: Bioeng Transl Med. 2026 Apr 13;11(4):e70145. doi: 10.1002/btm2.70145 (PMC13327607; doi:10.1002/btm2.70145)

## Slide 1
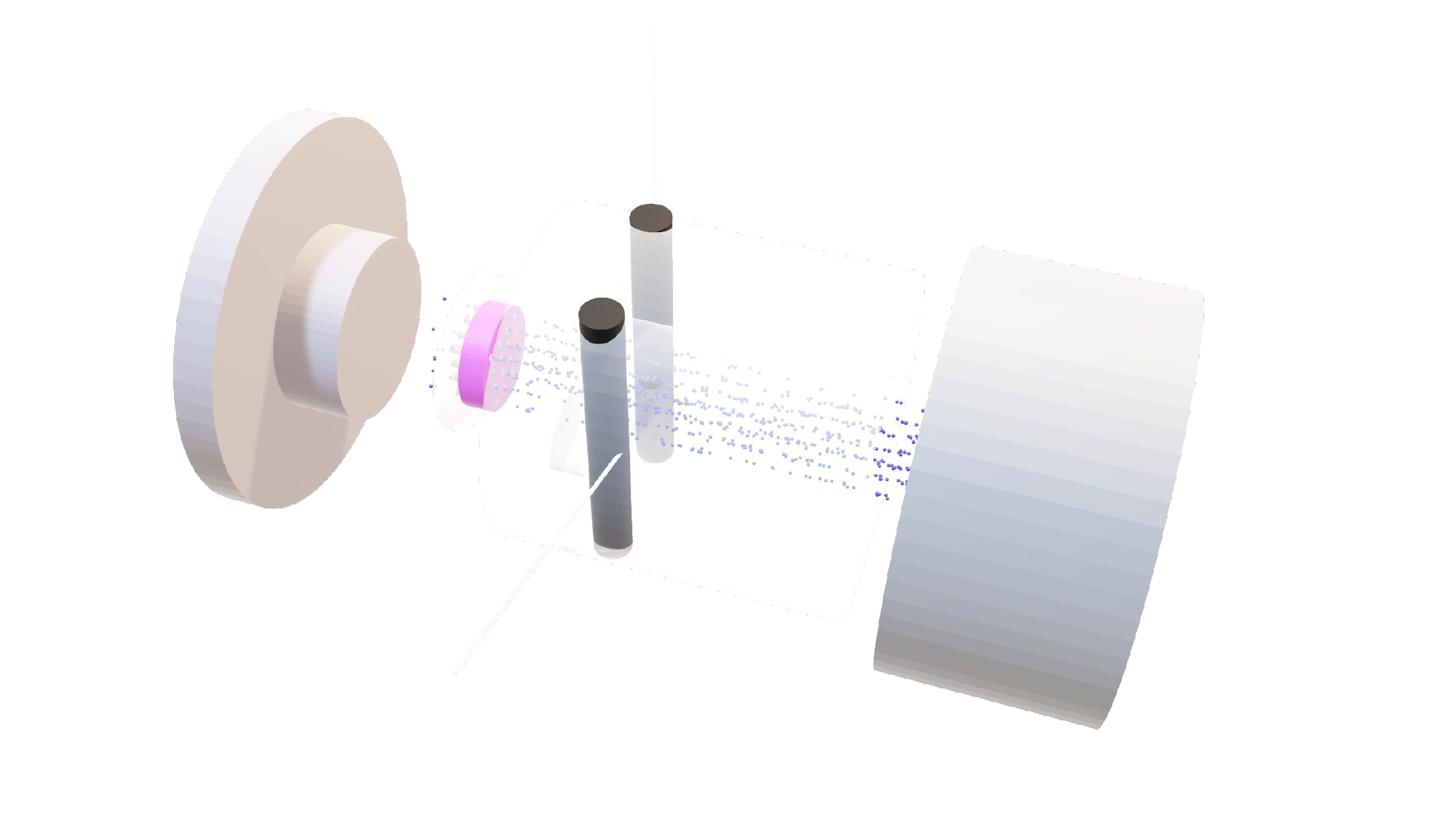

Supplement: Supplementary file 1 — APPENDIX S1: 3D‐software rendering of the complete bioreactor set‐up (explosion diagram). [file BTM2-11-e70145-s012.pptx]

## Slide 1
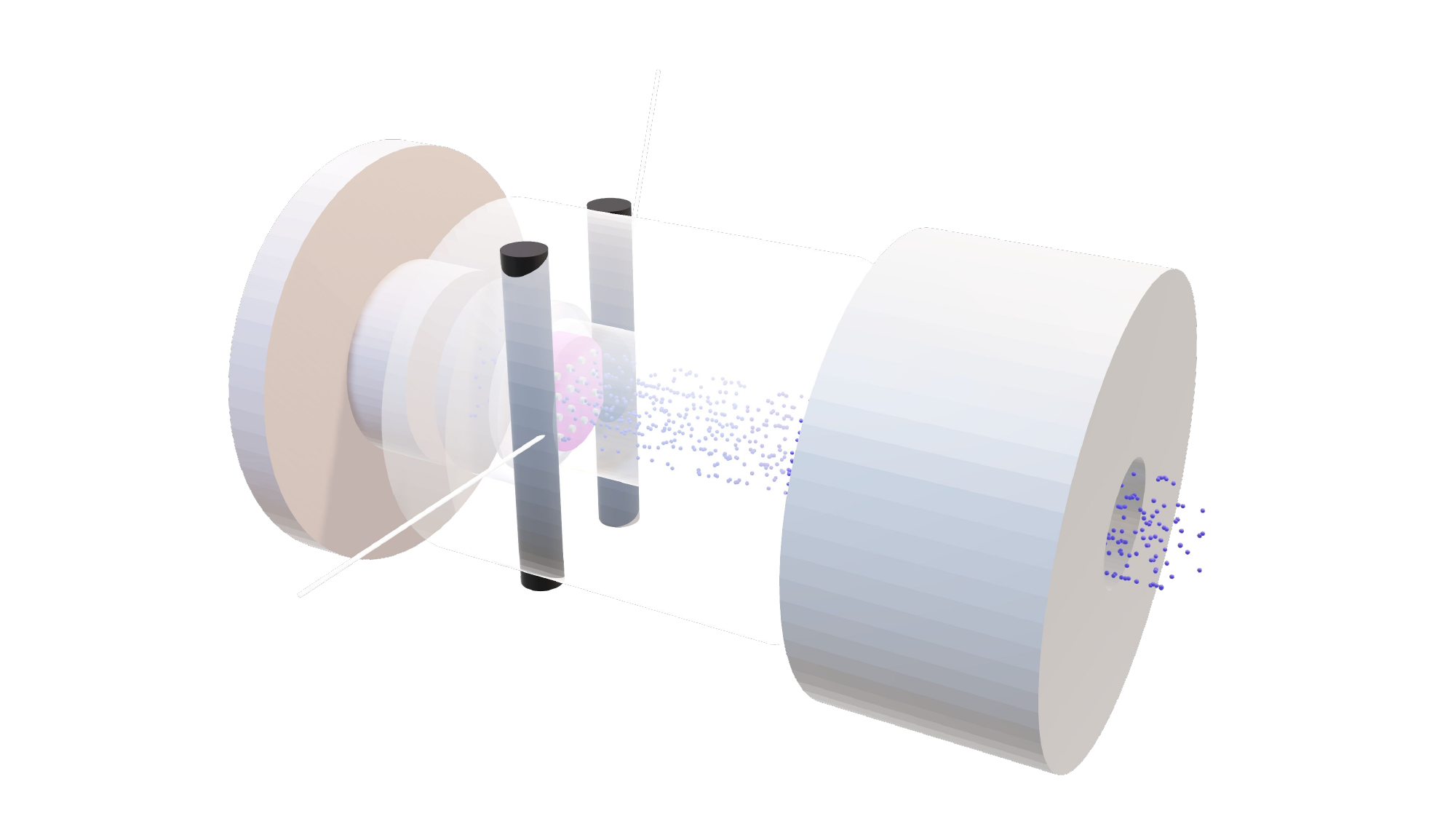

Supplement: Supplementary file 2 — APPENDIX S2: 3D‐software rendering of the complete bioreactor set‐up (non‐explosion diagram). [file BTM2-11-e70145-s010.pptx]

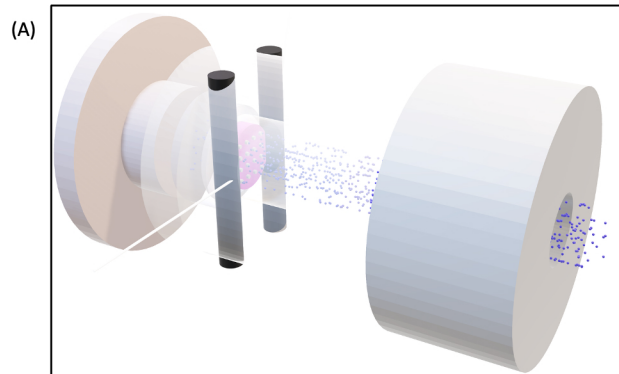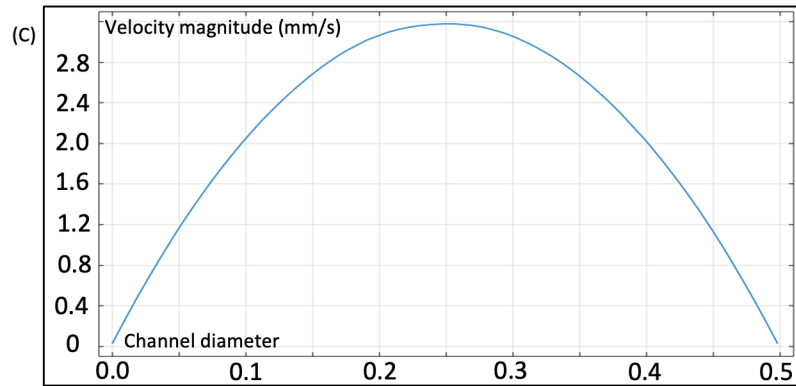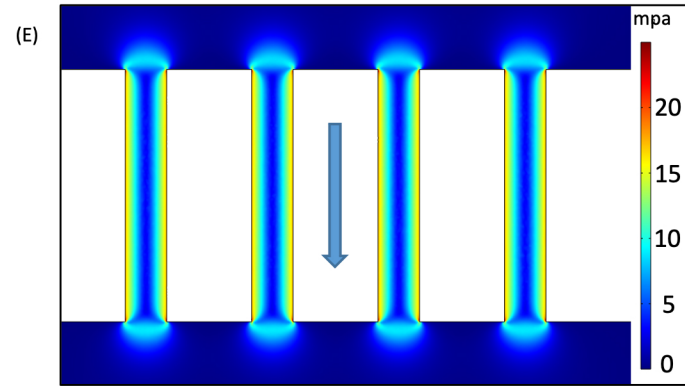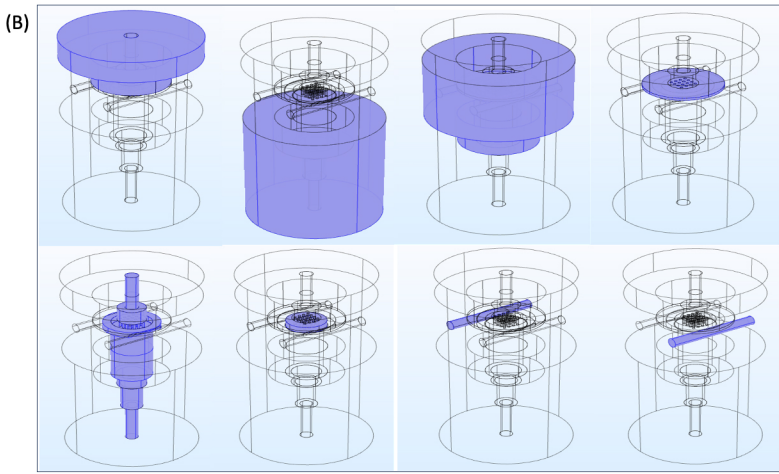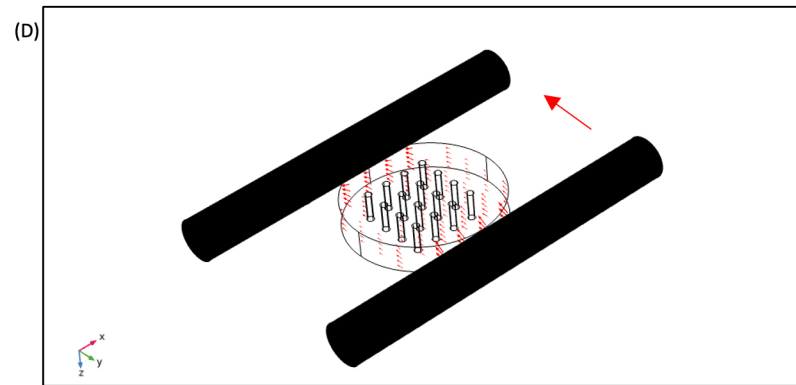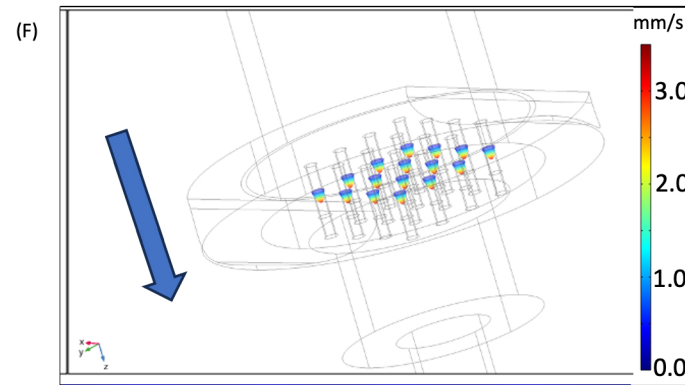

Supplement: Supplementary file 3 — FIGURE S1: Bioreactor design. (a) 3D model of the perfusion bioreactor. The hydrogel is in pink with visible channels. (b) Schematic representation of the bioreactor chamber set‐up. (c) Line graph of the velocity magnitude. (d) Current density simulation throughout the tissue construct horizontal plane. (e) Close up representation of wall shear stress inside the perfusion channels, in mPa. Arrow signals directions of fluid flow. (f) Close up representation of the channel perfusion velocity in mm/s. Arrow signals directions of fluid flow. [file BTM2-11-e70145-s007.pdf]

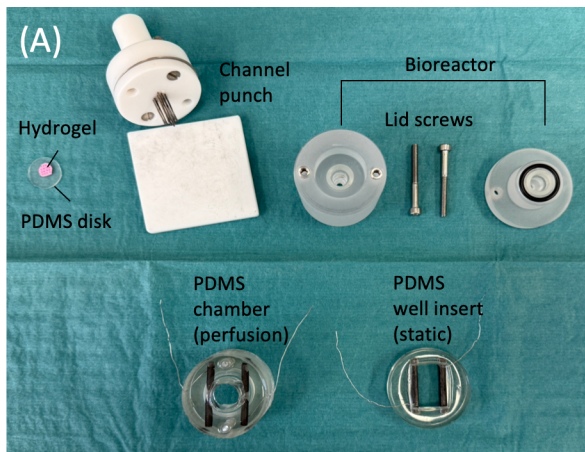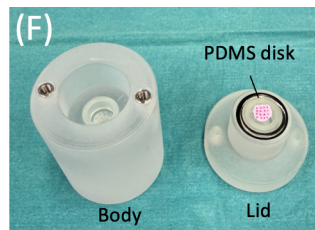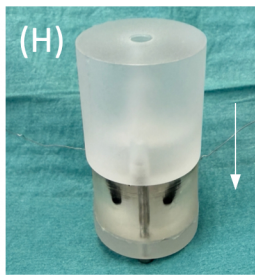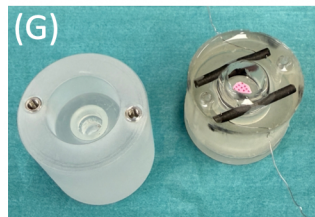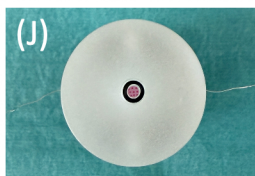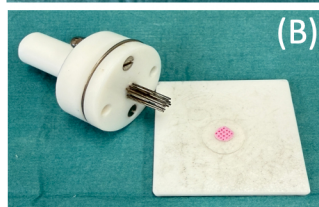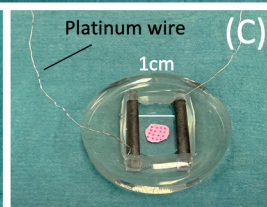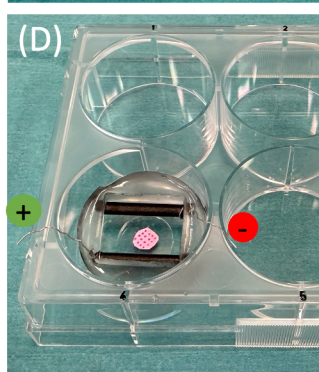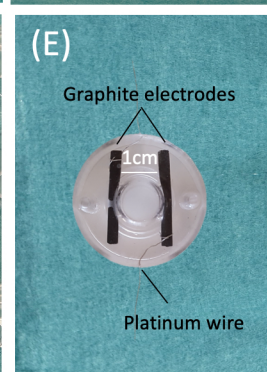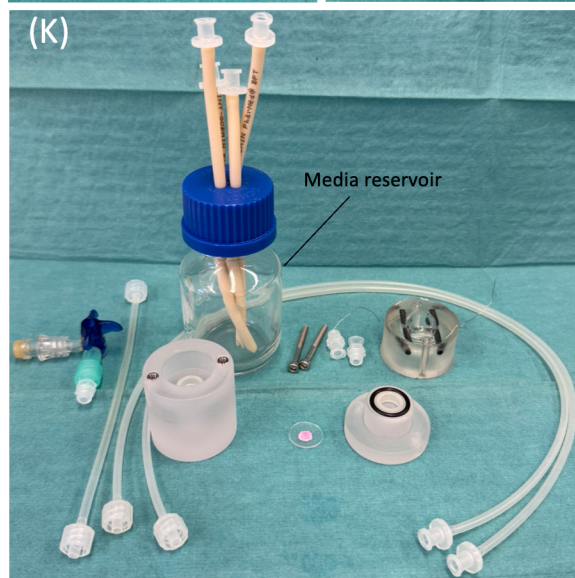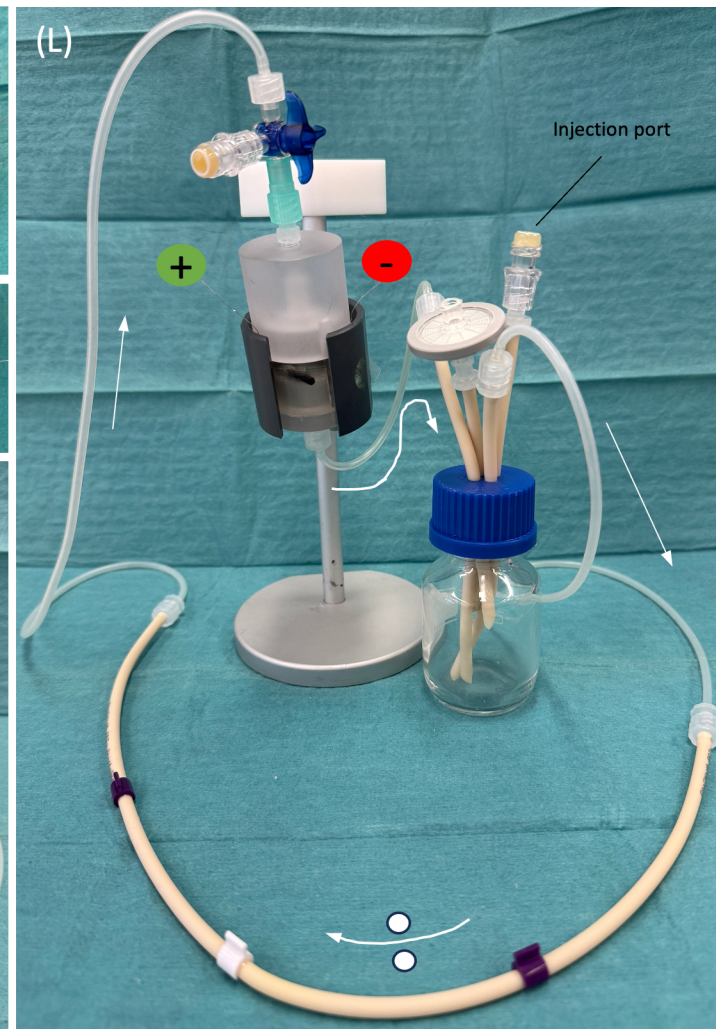

Supplement: Supplementary file 4 — FIGURE S2: Bioreactor set‐up. (a) Bioreactor assembly. (b) Channel punch. (c) PDMS well insert (static). (d) PDMS well insert, in position in a six‐well plate. The (+) and (−) indicate the electrical stimulation connection to the platinum wires. (e) PDMS bioreactor insert (perfusion). (f) PDMS disk in position with hydrogel atop. (g) PDMS bioreactor in position atop the bioreactor chamber lid. (h) Full mount with screws, side view. Arrow indicates media flow direction. (j) Bird's eye view inside the perfusion chamber with visible hydrogel. (k) Full perfusion set‐up with media‐reservoir and tubing. (l) Assembled perfusion bioreactor. The (+) and (−) indicate the electrical stimulation connection to the platinum wires. Arrows indicate media flow direction. The circles indicate positioning of roller pump. [file BTM2-11-e70145-s004.pdf]

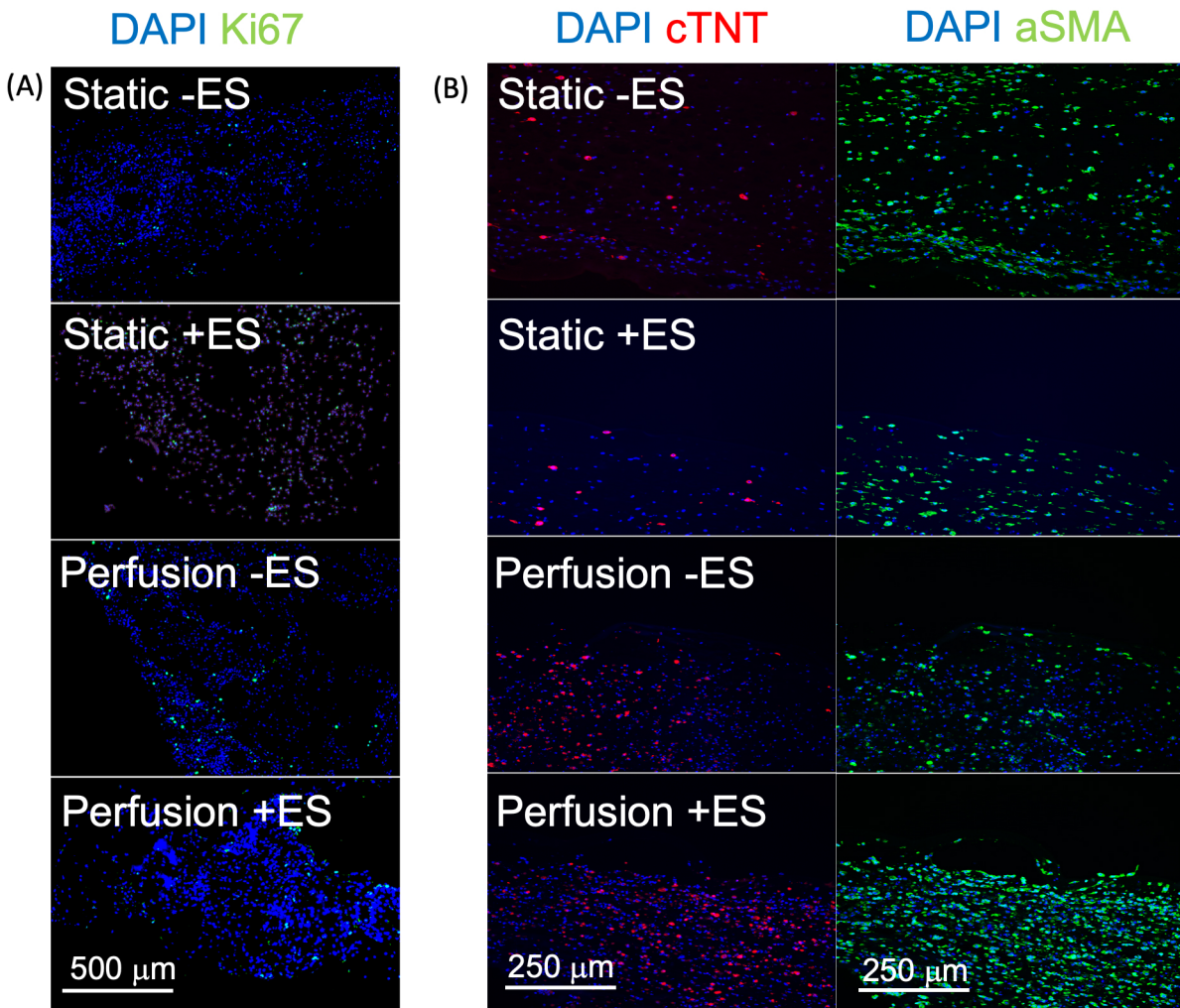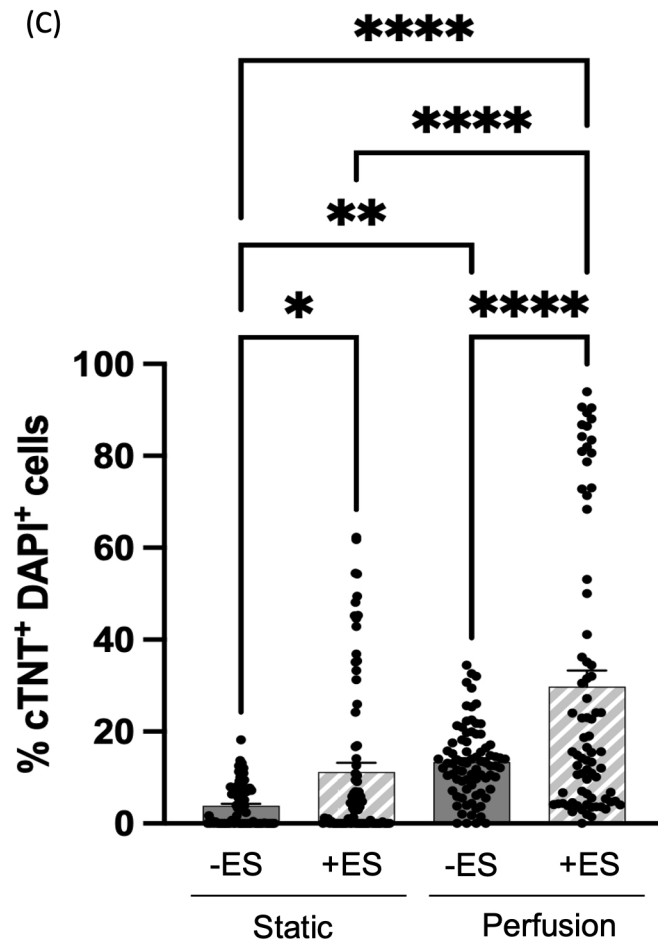

Supplement: Supplementary file 5 — FIGURE S3: Immunofluorescent analysis. (a) Immunofluorescent staining for DAPI in blue and Ki67 in green. (b) Immunofluorescent staining for DAPI in blue, cTNT in red and aSMA in green. (c) Quantification of cTNT+ and DAPI+ co‐localized cells. Data are represented as mean ± SEM. Comparisons were performed using a two‐way ANOVA and a Tukey's post‐hoc test (*p < 0.05, **p < 0.01, ****p < 0.0001). [file BTM2-11-e70145-s001.pdf]

***MyI2***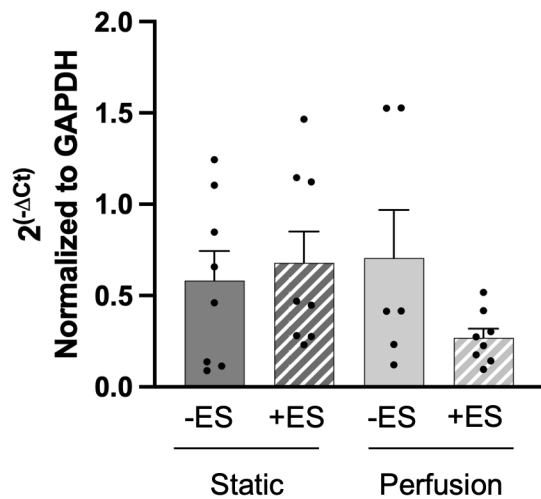***Myh6***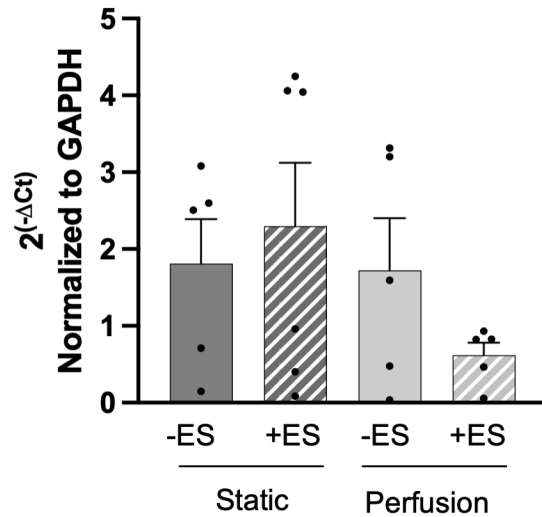***MyI7***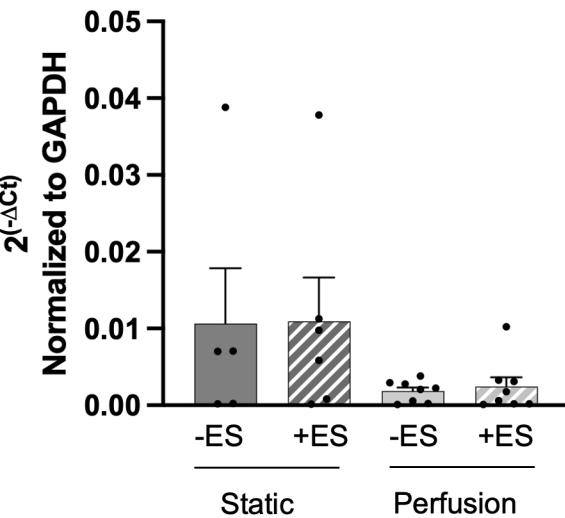***Myh7***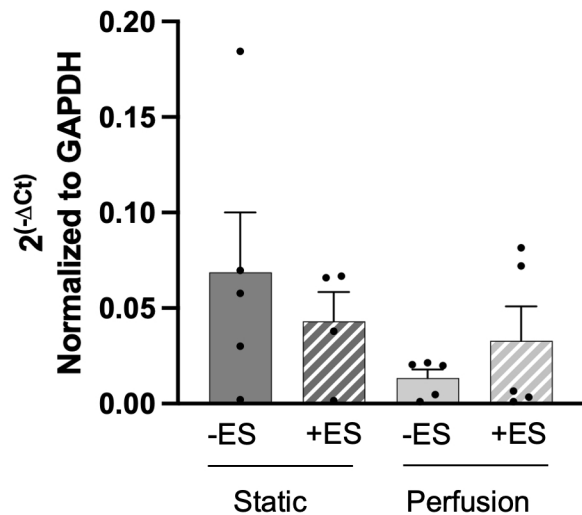

Supplement: Supplementary file 6 — FIGURE S4: Myosin chain analysis. Gene expression in RT‐PCR of relative increase of the myosin chain units: Myl2, Myl7, Myh6, Myh7. Comparisons were performed using a two‐way ANOVA test and normalized to Gapdh as a housekeeping gene. All data are represented as mean ± SEM. RT‐PCR analysis was performed on a minimum of four samples from a minimum of two independent experiments. [file BTM2-11-e70145-s006.pdf]

## Average pixel displacement

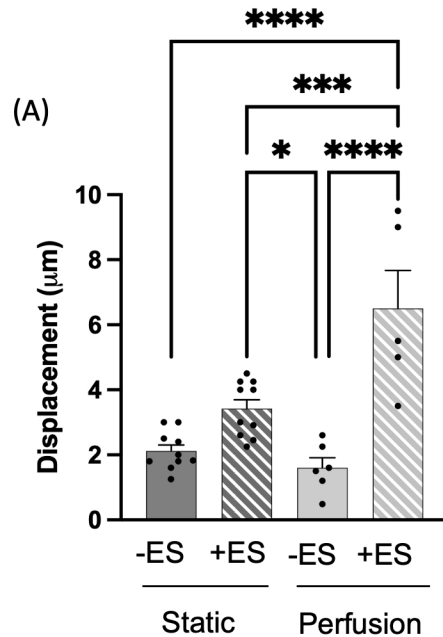

(B)

Relaxed state

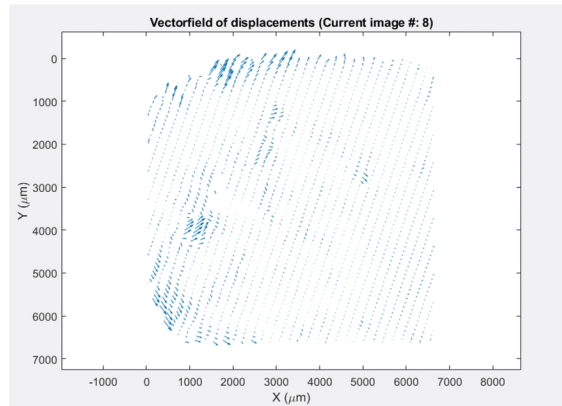

Mid-contraction

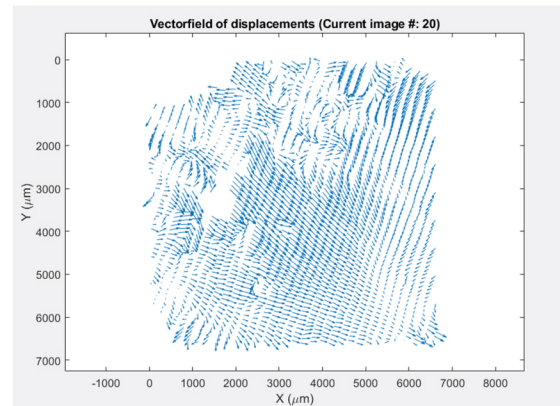

End-contraction

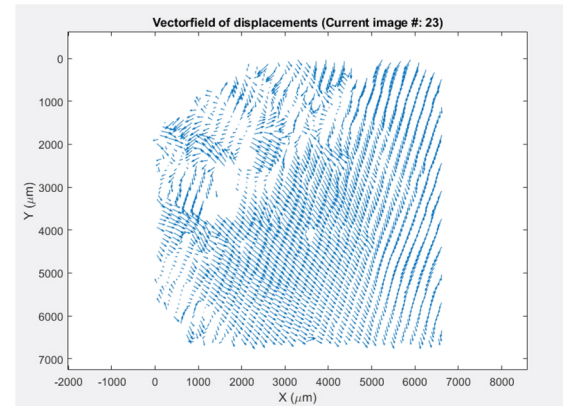

Supplement: Supplementary file 7 — FIGURE S5: Average pixel displacement. (a) Average pixel displacement of minimum and maximum displacement combined. Comparisons were performed using a two‐way ANOVA and a Tukey's post‐hoc test. (b) Vector map example for a perfusion + ES construct read‐out. All data are represented as mean ± SEM (*p < 0.05, ***p < 0.001, ****p < 0.0001). [file BTM2-11-e70145-s008.pdf]
