# Supplementary material for: Open‐source bioreactor delivers electrical and perfusion stimulation supporting 3D cardiac engineered tissue maturation
Source: Bioeng Transl Med. 2026 Apr 13;11(4):e70145. doi: 10.1002/btm2.70145 (PMC13327607; doi:10.1002/btm2.70145)
Supplement: Supplementary file 8 — TABLE S1: List of electrical conductivity (S/mm) and relative permittivity of materials used. References are given. [file BTM2-11-e70145-s002.docx]

**Supplementary table 1:**

|  | PDMS inset | Carbon rods | Culture medium | Polycarbonate | Hydrogel construct |
| --- | --- | --- | --- | --- | --- |
| Electrical Conductivity  (S/mm) | 0.83 x 10^-12 70^ | 1.28 x 10^6^ | 1.5^57^ | 1 x 10^-14^ | 1.5 |
| Relative permittivity | 2.69^71^ | 12 | 80^72^ | 3.1 | 80 |

List of electrical conductivity (S/mm) and relative permittivity of materials used. References are given.

Additional References:

70. Pavesi A, Soncini M, Zamperone A, Pietronave S, Medico E, Redaelli A, Prat M, Fiore GB. Electrical Conditioning of Adipose-Derived Stem Cells in a Multi-Chamber Culture Platform. *Biotechnol Bioeng*. 2014;111:1452-1463. doi:10.1002/bit.25201/abstract

71. 71. Tsai PJ, Nayak S, Ghosh S, Puri IK. Influence of particle arrangement on the permittivity of an elastomeric composite. *AIP Adv*. 2017;7(1). doi:10.1063/1.4973724

72. 73. Chen MT, Jiang C, Vernier PT, Wu YH, Gundersen MA. Two-dimensional nanosecond electric field mapping based on cell electropermeabilization. *PMC Biophys*. 2009;2(1). doi:10.1186/1757-5036-2-9
