# Supplementary material for: Open‐source bioreactor delivers electrical and perfusion stimulation supporting 3D cardiac engineered tissue maturation
Source: Bioeng Transl Med. 2026 Apr 13;11(4):e70145. doi: 10.1002/btm2.70145 (PMC13327607; doi:10.1002/btm2.70145)
Supplement: Supplementary file 9 — TABLE S2: Summary of the stimulation parameters, flow settings, and key findings for each experimental group. [file BTM2-11-e70145-s003.docx]

**Supplementary table 2:**

| **Experimental Group** | **Flow condition** | **Electrical Stimulation** | **Dapi^+^ cells/mm^2^** | **Mature CMs %** (cTNT^+^ αSMA^-^) | **Immature CMs %**  (cTNT^+^ αSMA^+^) | **Activated fibroblasts** % (cTNT^-^ αSMA^+^) | **Fibroblasts %** (cTNT^-^ αSMA^-^) |
| --- | --- | --- | --- | --- | --- | --- | --- |
| Static -ES | Static | None | 273.6 + 25.60 | 0.15 + 0.07 | 7.52 + 0.55 | 78.06 + 1.11 | 14.26 + 1.08 |
| Static +ES | Static | 3.5 V  (3 V/cm);  1Hz; 2ms | 303.3 + 33.14 | 0.29 + 0.14 | 22.21 + 3.03 | 66.75 + 2.40 | 10.75 + 1.37 |
| Perfusion -ES | Perfusion  (0.3 ml/min) | None | 553.6 + 65.12 | 14.39 + 1.26 | 12.31 + 1.251 | 13.43 + 0.89 | 59.86 + 2.13 |
| Perfusion +ES | Perfusion  (0.3 ml/min) | 6.08 V  (3 V/cm);  1 Hz; 2 ms | 640.8 + 68.03 | 8.11 + 1.0 | 51.44 + 4.90 | 32.90 + 4.60 | 7.55 + 1.19 |
| **Experimental Group** | **Flow condition** | **Fibroblasts %** (cTNT^-^ αSMA^-^) | **Cardiomyocyte length-width ratio** | **Average sarcomere length** | **Excitation threshold** **(ET)** | **Maximum capture rate (MCR)** | **average pixel displacement (APD)** |
| Static -ES | Static | 14.26 + 1.08 | 1.775 + 0.09 | 0.89 + 0.03 | 6.5 + 0.37 | 3.0 + 0.17 | 4.04 + 0.46 |
| Static +ES | Static | 10.75 + 1.37 | 2.74 + 0.21 | 0.96 + 0.03 | 4.8 + 0.31 | 3.9 + 0.18 | 5.53 + 0.39 |
| Perfusion -ES | Perfusion  (0.3 ml/min) | 59.86 + 2.13 | 1.70 + 0.13 | 0.79 + 0.01 | 6.0 + 0.42 | 3.5 + 0.19 | 2.79 + 0.66 |
| Perfusion +ES | Perfusion  (0.3 ml/min) | 7.55 + 1.19 | 2.43 + 0.12 | 1.06 + 0.03 | 2.2 + 0.16 | 4.2 + 0.24 | 13.36 + 1.61 |

Summary of the stimulation parameters, flow settings, and key findings for each experimental group.
